# A convenient correspondence between *k*-mer-based metagenomic distances and phylogenetically-informed *β*-diversity measures

**DOI:** 10.1371/journal.pcbi.1010821

**Published:** 2023-01-06

**Authors:** Hongxuan Zhai, Julia Fukuyama

**Affiliations:** Department of Statistics, Indiana University Bloomington, Bloomington, Indiana, United States of America; Fudan University, CHINA

## Abstract

*k*-mer-based distances are often used to describe the differences between communities in metagenome sequencing studies because of their computational convenience and history of effectiveness. Although *k*-mer-based distances do not use information about taxon abundances, we show that one class of *k*-mer distances between metagenomes (the Euclidean distance between *k*-mer spectra, or EKS distances) are very closely related to a class of phylogenetically-informed *β*-diversity measures that do explicitly use both the taxon abundances and information about the phylogenetic relationships among the taxa. Furthermore, we show that both of these distances can be interpreted as using certain features of the taxon abundances that are related to the phylogenetic tree. Our results allow practitioners to perform phylogenetically-informed analyses when they only have *k*-mer data available and provide a theoretical basis for using *k*-mer spectra with relatively small values of *k* (on the order of 4-5). They are also useful for analysts who wish to know more of the properties of any method based on *k*-mer spectra and provide insight into one class of phylogenetically-informed *β*-diversity measures.

## Author summary

Microbiologists have two major strategies for understanding the bacterial communities present in the environment: shotgun metagenome sequencing and amplicon sequencing. Both involve taking samples from the environment, extracting DNA from those samples, and sequencing the extracted DNA. They have different strengths and give different kinds of information about the communities. Because they give different kinds of information, methods for analyzing microbiome data tend to be developed for and used on just one kind of study. In this paper, we show a strong relationship between a set of methods for measuring distances between samples in shotgun metagenome sequencing datasets (the *k*-mer-based distances) and a set of methods for measuring distances between samples in amplicon sequencing datasets (the phylogenetically-informed beta diversity measures). This is a convenient correspondence because *k*-mer spectra are easier to extract from shotgun metagenome sequencing datasets than the taxon abundances that would be needed to compute the phylogenetically-informed *β* diversities. Therefore, if an analyst would like to compute phylogenetically-informed distances between communities from a shotgun metagenome sequencing dataset, our results show that they can work directly with the *k*-mer spectra and not worry about estimating taxon abundances. The results also imply that any of the many methods that are based on *k*-mer spectra are implicitly using phylogenetic information.

This is a *PLOS Computational Biology* Methods paper.

## 1 Introduction

Although we have known for at least a century about the microorganisms that live in and around us, only in the past several decades have we had the tools to investigate these microbial communities in a high-throughput and relatively unbiased way. In particular, high-throughput DNA sequencing technologies have provided a lens that allows us to investigate the bacteria present in the environment.

To go from short sequences taken from environmental DNA to a description of the bacteria present in a sample, investigators have taken one of two main approaches: they either focus on a marker gene representing just a small part of the genome of each of the bacterial taxa, or they perform shotgun metagenome sequencing in which they take a sample of short sequences from across the entire genomes of all the bacteria present in the sample. In marker gene studies, the sequence of the marker gene is used as a proxy for bacterial species, and the relative abundances of each marker gene sequence correspond to the abundance of the corresponding species in the sample. In shotgun metagenome sequencing studies, analysts aim to assemble entire bacterial chromosomes from the short reads. If this were done perfectly, we would be able to use the assembled chromosomes to see the bacterial genomes present in each sample and use the genomes to infer which bacterial taxa are present in each sample and what their relative abundances are. Unfortunately, assembly is very difficult: a unique solution does not usually exist even in single-genome assembly [1], and the problem is exacerbated in metagenome assembly by the possibility of nearly identical sequences in different community members.

In either case, a common task is to compare the samples to each other so as to make inferences about the differences or similarities in the microbiota in different environments or experimental conditions. In marker gene studies a taxon abundance matrix is available, and distances between samples can be measured using standard ecological measures of difference, such as the Bray-Curtis dissimilarity [2] or the Jaccard index [3]. A variant on the standard measures are phylogenetically-informed distances or phylogenetically-informed *β*-diversity measures, which are supposed to measure the dissimilarities between microbial communities while taking into account the evolutionary relationships among the taxa present in those communities. Several such measures have been proposed [4–7], and we will focus here on a certain class of distances (the modulated phylogenetically-informed distances based on Rao’s quadratic entropy, or MPQ Distances) that have been previously described for microbiome data [8] and which are based on Rao’s quadratic entropy [9].

A different approach is usually taken for defining distances among samples in shotgun sequencing datasets. Although it is possible to use one of the standard ecological indices on the contig abundances (the stretches of assembled sequences that one creates from shotgun sequencing data), many of the reads remain unassembled in these studies, and analysts will often use *k*-mer-based distances instead since they allow for the use of all the reads. The idea is to count the number of times each unique *k*-mer appears in each of the metagenome samples and use the resulting *k*-mer spectra to quantify dissimilarities between samples. Other groups have shown that when *k* is large, on the order of 30, these dissimilarities match the standard, non-phylogenetic ecological distances that they would have gotten from the taxon abundance information [10–13]. Since there are about 10^18^ 30-mers, the naive approach to computing these spectra would be an enormous computational undertaking, but clever design strategies keep the task from being intractable [14–16]. In contrast, we will see that smaller values of *k* lead to dissimilarities that match some of the existing phylogenetically-informed *β*-diversity measures.

**Fig 1:**
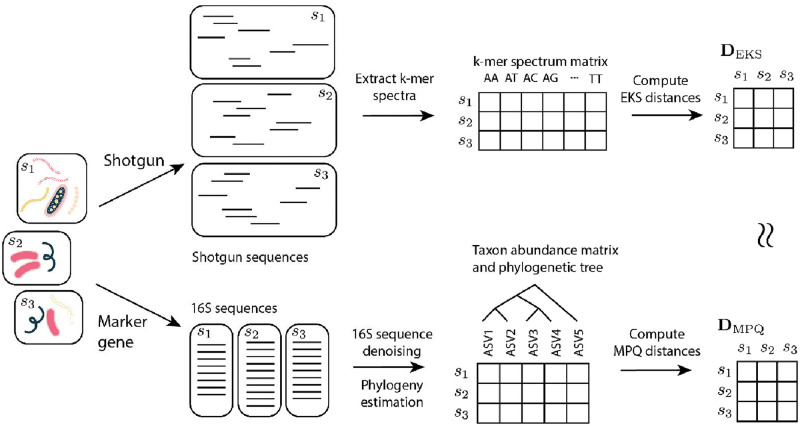
Schematic of the process for computing MPQ distances and EKS distances. We show that there is a close relationship between the MPQ distances, which are designed for marker gene studies, and the EKS distances, which are designed for shotgun metagenome sequencing studies.

**Fig 2:**
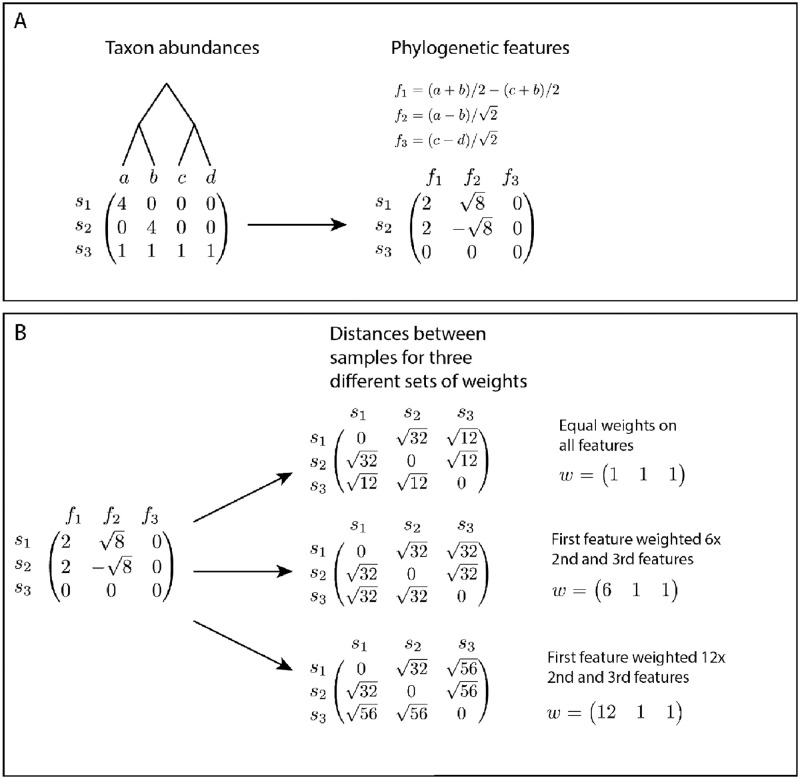
(A) An example of computing phylogenetically-informed features *f*_1_, *f*_2_, and *f*_3_ on a taxon abundance matrix. (B) Computing a weighted Euclidean distance between samples *s*_1_, *s*_2_, and *s*_3_ using the features defined in (A) and equal weights for the three features (top row), and increased weight on the first feature relative to the second and third features (middle and bottom rows). As we increase the weight on *f*_1_, the distance between *s*_3_ and the other samples increases compared to the distance between *s*_1_ and *s*_2_.

In this paper, we investigate the relationship between phylogenetically-informed *β*-diversity measures and *k*-mer-based metagenomic distances. We specialize to the Euclidean distances on *k*-mer spectra (EKS distances) and a set of modulated phylogenetic distances based on Rao’s quadratic entropy (MPQ distances). The difficulty in comparing these methods comes from the fact that they use two different kinds of data, but we will show empirically and theoretically that they lead to similar sets of distances among the samples (Fig 1). Along the way, we show that the reason the two sets of distances are so similar is that they can both be interpreted as creating a new set of features from the (unobserved in the shotgun sequencing case) taxon abundances in each of the samples and computing a weighted Euclidean distance on the new set of features (Fig 2). Finally, we note that the focus here is on small values of *k*. We will see that as *k* increases, distances between *k*-mer spectra become closer to the standard Euclidean distance between taxon abundance vectors and the phylogenetic information is lost. These results give us a solid theoretical reason to use *k*-mer spectra with relatively small values of *k* for performing phylogenetically-informed analyses, allowing practitioners to skip the assembly, mapping, and phylogenetic assingment steps when performing phylogenetically-informed tests or ordinations on shotgun sequencing data.

## 2 Approach

We will demonstrate the relationship between these two kinds of measures through a combination of theoretical results and computational experiments. Our approach here is to show that the Euclidean distance between the *k*-mer spectra corresponding to two metagenomes can be re-expressed as a weighted Euclidean distance on a set of linear features of the unobserved taxon abundances corresponding to those metagenomes, and that those features are highly related to the phylogenetic tree. We also show that the MPQ distances have exactly the same interpretation as a weighted Euclidean distance on a set of linear features of the taxon abundances. (See Fig 2 for an example of what these features look like and how they can be used to compute distances between samples.) The definition of these features depends on the phylogenetic tree relating the taxa. In a simple case, when the phylogenetic tree is a balanced binary tree, the features and relative weights implicitly used by the EKS distances and the MPQ distances are exactly the same. In more complex situations, we show empirically that the features corresponding to the EKS distances are similar to those for the MPQ distances, and we show in real data sets that the representations of the samples given by the two methods are similar.

## 3 Results

### 3.1 Relationships between *k*-mer spectra are equivalent to relationships between taxon abundances in a non-standard inner product space

**Fig 3:**
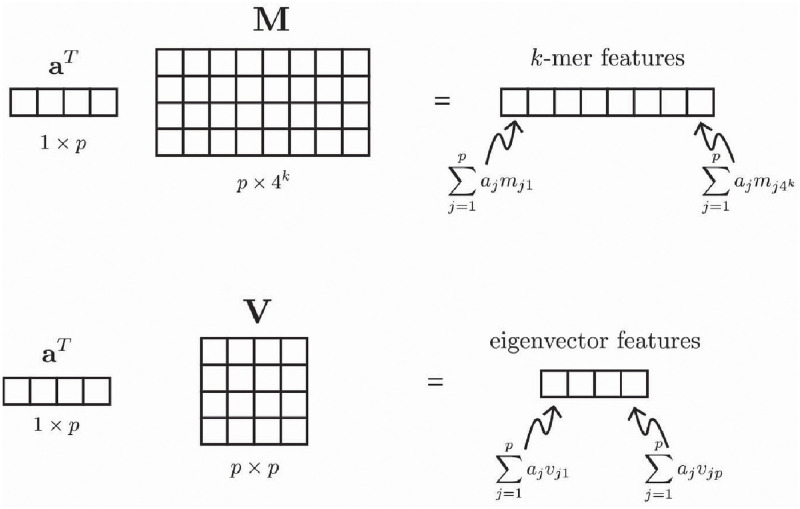
Schematic of how *k*-mer features (top) and eigenvector features (bottom) are computed.

The fact that allows us to compare phylogenetically-informed *β*-diversity measures, which are based on taxon abundances, and *k*-mer-based distances, which are based on *k*-mer spectra, is that there is a linear map from taxon abundances to *k*-mer spectra. We will call this map **M**. It can be expressed as a *p* × 4^*k*^ matrix, where *p* is the number of taxa and 4^*k*^ is the potential number of unique *k*-mers, with element *m*_*ij*_ containing the number of times the *j*th *k*-mer appears in the *i*th taxon. See Fig 3, top row for an illustration.

A consequence of this fact is that any method based on the Euclidean distance between *k*-mer spectra will be equivalent to a method that works with the taxon abundances in a non-standard inner product space. Suppose that **a**^*i*^ is a length-*p* vector representing the abundances of the *p* taxa in the *i*th sample. The non-standard inner product space in question will be one for which instead of having the inner product between two taxon abundance vectors **a**^1^ and **a**^2^ be 〈**a**^1^, **a**^2^〉 = (**a**^1^)^*T*^ **a**^2^, we use ${\langle a^{1},a^{2}\rangle }_{MM^{T}}={\left(a^{1}\right)}^{T}MM^{T}a^{2}$. The squared distance between **a**^1^ and **a**^2^ in this space is *d*(**a**^1^, **a**^2^)^2^ = (**a**^1^, **a**^2^)^*T*^ **MM**^*T*^ (**a**^1^, **a**^2^). Since (**a**^1^)^*T*^ **M** is the *k*-mer spectrum corresponding to the abundances in **a**^1^, and similarly (**a**^2^)^*T*^ **M** is the *k*-mer spectrum corresponding to the abundances in **a**^2^ (see Fig 3, top row), we see immediately that in this inner product space, the distance between abundance vectors is the same as the Euclidean distance between *k*-mer spectra (which would be [(**M**^*T*^ **a**^1^ − **M**^*T*^ **a**^2^)^*T*^ (**M**^*T*^ **a**^1^ − **M**^*T*^ **a**^2^)]^1/2^).

### 3.2 Features implicit in the inner product space are given by the eigendecomposition of MM^*T*^

Next, notice that if **Q** is the matrix defining the inner product, the eigendecomposition of **Q** gives an interpretation of distances in this inner product space in terms of a weighted Euclidean distance between a certain set of features derived from the taxon abundance vectors. Specifically, if **Q** = **VΛV**^*T*^ is the eigendecomposition of **Q** such that $V\in R^{p\times p}$ is orthogonal and $\Lambda \in R^{p\times p}$ is diagonal, then the columns of **V** give the features and the diagonal elements in **Λ** give the squared weights. The idea is that each of the features defined by a column of **V** is a linear combination of the taxon abundances, so that the value of the *j*th feature for a set of taxon abundances given in the taxon abundance vector **a** is given by $\sum_{i=1}^{p}a_{i}v_{ij}$. See Fig 3, bottom row, for an illustration.

We can see that this featurization is analogous to the featurization provided by **M**: as with the eigendecomposition, the columns of **M** define features (in the case of **M**, the number of times the relevant *k*-mer is present in each of the taxa), and all of the features have equal weight. One can see that many featurizations are possible, but the advantage of the eigendecomposition for interpretation is that the features provided are non-redundant (linearly independent), and, aside from features with the same weight, unique. Features provided by arbitrary featurizations such as the *k*-mer description provided by **M** are harder to interpret because of the potential redundancies: features carrying the same information could be spread out across the columns of **M** (the columns can be linearly dependent).

With the featurization defined by the eigendecomposition, the distance between **a**^1^ and **a**^2^ can then be expressed as a weighted Euclidean distance between (**a**^1^)^*T*^ **V** and (**a**^2^)^*T*^ **V** (the set of features corresponding to **a**^1^ and the set of features corresponding to **a**^2^). The weights in the weighted Euclidean distance are the square roots of the diagonal elements of Λ.

For the distance between *k*-mer spectra, the matrix defining the inner product is **MM**^*T*^. Because the features given by the eigendecomposition of **MM**^*T*^ provide a nice interpretation of the inner product space, we will investigate the eigendecomposition. Since we are interested in how **MM**^*T*^ is related to the tree joining the taxa it describes, and since this matrix is random and will be different for every study, we investigate the expected value, *E*(**MM**^*T*^). If we have *p* taxa, we will fix a tree structure with *p* leaves, invoke a model of sequence evolution that proceeds along the fixed tree structure, and investigate *E*(**MM**^*T*^) in this model. We will see that the features given by the eigenvectors are highly related to the phylogenetic tree that we use. This result shows that *k*-mer-based distances are implicitly phylogenetically-informed *β*-diversity measures, and, at least in simple cases, we can give the exact form of the phylogenetic features.

### 3.3 The eigenvectors for the balanced binary tree are contrasts between sister clades

We start our investigation with a simple case, where the tree structure is a balanced binary tree and we assume that the sequences have evolved on the tree according to the model of sequence evolution described by Jukes and Cantor [17]. It turns out that in this case, we can show that *E*(**MM**^*T*^) has a certain kind of block structure (see Section 1 of S1 Text).

**Fig 4:**
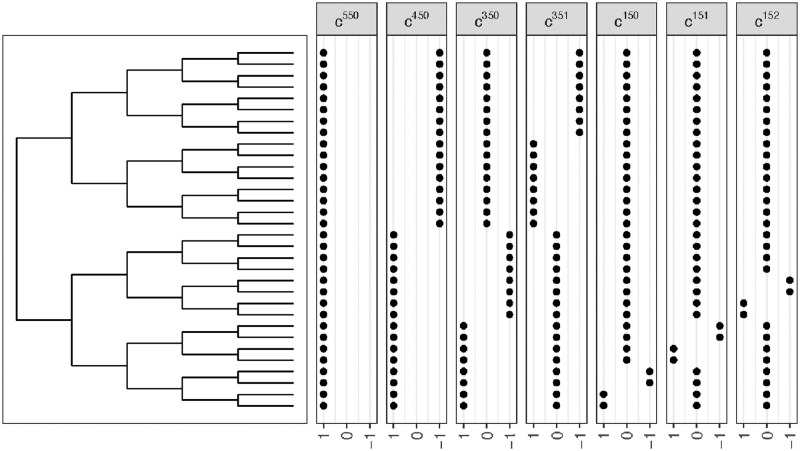
Illustration of the vectors c^*ijk*^ for *j* = 5 and various values of *i*.

We can leverage the block structure along with some other properties of *E*(**MM**^*T*^) to obtain its eigenvalues and eigenvectors, but we first need some notation to describe the structure of the matrix and the eigenvectors. Let **D**^*ij*^ denote a 2^*j*^ × 2^*j*^ matrix with 2^*i*^ × 2^*i*^ blocks of 1’s on the diagonals. Let **c**^*ijk*^ denote a vector of length 2^j^ with a block of 2^*i*+1^ non-zero elements, with the first 2^i^ of the non-zero elements equal to 1 and the second block of 2^i^ non-zero elements equal to −1. For fixed values of *i* and *j*, we have 2^*j*−*i*−1^ possibilities for *k* (see Fig 4 for an illustration).

We can write (see the S1 Text for a proof):

$$\begin{array}{c} E\left(MM^{T}\right)=\sum_{i=0}^{j-1}\left(\mu_{i}-\mu_{i+1}\right)D^{ij}+\mu_{j}D^{jj} \end{array}$$

for some *μ*_0_, …, *μ*_*j*_ > 0. Then the **c**^*ijk*^ vectors are the eigenvectors of *E*(**MM**^*T*^), and the eigenvalue corresponding to eigenvector **c**^*ijk*^ is

$$\begin{array}{c} \lambda_{i}=\sum_{i^{\prime }=0}^{i}2^{i^{\prime }}\left(\mu_{i^{\prime }}-\mu_{i^{\prime }+1}\right) \end{array}$$ (1)

Notice that *E*(**MM**^*T*^) is a sum of block diagonal matrices and that *μ*_*i*_ is the value of the elements of *E*(**MM**^*T*^) that are covered by a block of size 2^*i*^ but not smaller blocks. That is, *μ*_0_ is the value on the diagonal, *μ*_1_ is the value on portions of the matrix that are 1 away from the diagonal, *μ*_2_ is the value on the portions of the matrix that are more than 2^1^ − 1 = 1 but no more than 2^2^ − 1 = 3 away from the diagonal, and so on. The expression for the eigenvalues tells us that so long as *μ*_*i*_ ≥ *μ*_*i*+1_∀*i*, **c**^*jj*0^ is the eigenvector with the largest eigenvalue, **c**^*j*−1,*j,k*^ are the two eigenvectors with second-largest eigenvalue, **c**^*j*−2,*j,k*^ are the four eigenvectors with the third largest eigenvalue, and so on. Because **MM**^*T*^ is a similarity matrix, we expect that *μ*_*i*_ > *μ*_*i*+1_, but we do not have a proof. In simulations this inequality has always held.

Returning to our interpretation of eigenvectors as features and eigenvalues as weights for those features, this result tells us what the features are if the taxa are related to each other by a balanced binary tree. The feature with the largest weight is constant and represents an average across all the taxa (Fig 4, first column of features). The feature with the largest weight after that measures the difference in abundance between taxa in one half of the tree vs. the other (Fig 4, second column of features). The features with the highest weight after that measure the difference in abundance between taxa corresponding to one clade of size *p*/4 and its sister clade (Fig 4, third and fourth columns of features). Subsequent features with progressively lower weights measure the difference in abundance between successively smaller clades and their sisters (e.g. Fig 4, last three columns of features). Notice that these eigenvectors are exactly the same as Felsenstein’s phylogenetically independent contrasts [18]. Thus we see that in the case of the balanced binary tree, the features implied by the use of the *k*-mer spectra have a simple interpretation in terms of the phylogeny.

Although we have an expression for the eigenvalues for the balanced binary tree (Eq 1), we do not know the values of *μ*_0_, *μ*_1_, … in that expression. Therefore, to inestigate the eigenvalues, we use a Monte Carlo approximation to *E*(**MM**^*T*^) as described in Section 5.4.

**Fig 5:**
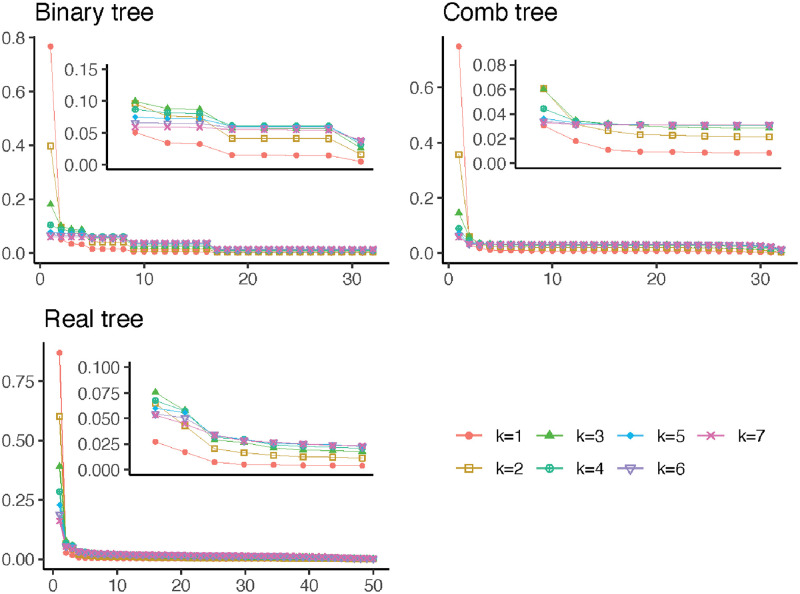
Eigenvalues for the matrix *E*(MM^*T*^) for the balanced binary tree, the comb tree, and the real data tree. The inset plots are the same as the main plots, but with the horizontal axis truncated to remove the first eigenvalue.

We see the eigenvalues for this balanced binary tree in the first panel of Fig 5. Recall that the eigenvalue corresponding to an eigenvector gives the weight for that eigenvector feature. The figure shows us that when *k* is small, the largest eigenvectors have relatively more weight than they do when *k* is large. This means that when *k* is small, the feature corresponding to the second eigenvector (a contrast between one half of the tree and the other) has much more weight than the feature corresponding to the third or fourth eigenvector (a contrast between a clade consisting of one quarter of the tree and its sister). When *k* is large, the feature corresponding to the second eigenvector still has more weight than the feature corresponding to the third or fourth eigenvectors, but the difference in weights is not nearly as pronounced.

### 3.4 The eigenvectors for the comb tree are smooth on the leaves of the tree

We next turn to the eigenvectors of *E*(**MM**^*T*^) for the comb tree, which in some sense is as different as possible from the balanced binary tree. We will see that in this case, the eigenvectors are not the same as they were for the balanced binary tree, but they are still “smooth” on the leaves of the tree and have an interpretation as phylogenetically informed features.

**Fig 6:**
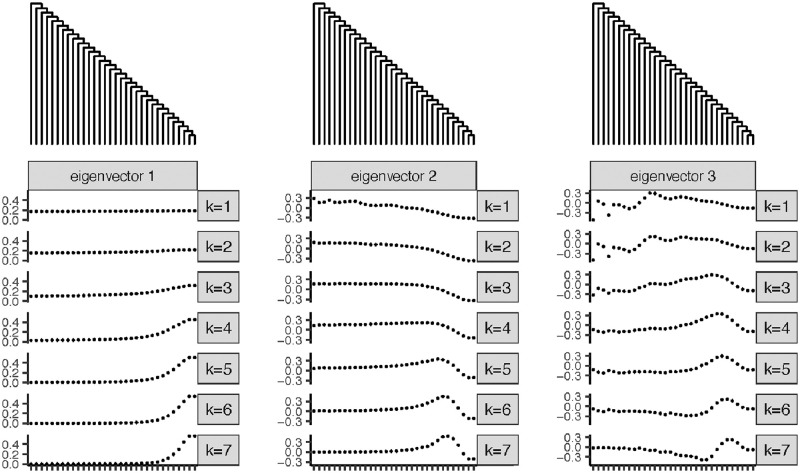
(Comb tree) The first, second and third-largest eigenvectors for different *k* values.

If the balanced binary tree is the most balanced type of tree, the comb tree is the least balanced type of tree. A comb tree is shown in Fig 6, where we see that at each node, one daughter has no further descendants and the other daughter is allowed to branch. There are a number of possibilities for the branch lengths in a comb tree, but here we will investigate an ultrametric comb tree.

For the comb tree with *p* leaves, we can show that *E*(**MM**^*T*^) is of the form

$$\begin{array}{c} \sum_{i=1}^{p}\left(e_{i}a_{i}^{T}+a_{i}e_{i}^{T}\right), \end{array}$$

where ***e***_*i*_ is the *i*th standard basis vector and ***a***_*i*_ is the column vector with entries from *a*_1_ to *a*_*i*−1_ equal to 0; the *i*th entry equal to *μ*_0_/2; the other entries equal to *μ*_*i*_.

Once we have this result, we can obtain the eigenvectors for the comb tree numerically as described in Section 5.4. We see the results in Fig 6. As before, the eigenvectors are smooth on the tree and have interpretations as features of the tree. In this case, the sequences are of length 7 and we compute the eigenvectors for *k* = 1, …, 7. We use this relatively short sequence so that we can see the eigenvectors for the full range of *k* relative to the sequence length.

To understand the eigenvectors corresponding to the comb tree, recall that for the balanced binary tree, the eigenvectors with the largest eigenvalues corresponded to clades that were both large and highly distinct (meaning that the elements of the clade are uniformly far away from their closest neighbors). For the ultrametric comb tree, these two criteria are in conflict with each other: the largest clade of size *n* is the set of the *n* taxa that diverge most recently from their neighbors. As a result, the eigenvectors are combinations of features corresponding to taxa that are very different from their closest neighbors and features corresponding to large clades.

The situation is different for *k* less than half the sequence length (*k* = 1, 2, 3) and *k* greater than half the sequence length (*k* = 4, …, 7), and so we discuss them separately. For *k* = 1, 2, 3, the first eigenvector is approximately an average across all the taxa. The second eigenvector can be described as a combination of two wavelets: one that is high frequency and that has largest amplitude for the left half of the tree where the taxa are most distinct, and one that is low frequency and has largest amplitude to the right of the tree where the taxa are least distinct. This phenomenon can be seen in Fig 6, where the high-frequency part of the feature is most clearly visible at the left of the tree for the second and third eigenvectors and *k* = 1. It can be seen more clearly in a tree with more leaves, shown in Fig B in S1 Text.

For *k* ≥ 4, we have wavelets focused on the right side of the tree. The first eigenvector is approximately an indicator of the three or four right-most taxa, the second is approximately a contrast between those taxa and their three or four closest neighbors, and so on. Overall, it seems that for larger values of *k*, the focus of the features is more on large clades of closely-related taxa, and for the smaller values of *k*, the focus is on a combination of large clades of closely-related taxa and smaller clades of highly distinct taxa.

The eigenvalues, giving the weights of the eigenvector features, show the same pattern for the comb tree as for the balanced binary tree (see Fig 5, second panel for the eigenvalues for the comb tree). When *k* is small, there is an enormous difference between the weight given to the top eigenvectors and the subsequent ones, and as *k* increases, the differences in the weights decrease. This means that for small *k*, the overwhelming majority of the weight is on the top couple of eigenvectors, but when *k* is larger the weights are spread out over a larger number of eigenvectors.

### 3.5 The eigenvectors for a tree created from real data are smooth on the tree

To demonstrate that the smoothness of the eigenvectors on the leaves of the tree in the cases of the balanced binary tree and the comb tree was not just a result of the regularity of those trees, we look at the eigenvectors for *E*(**MM**^*T*^) using a tree taken from a real microbiome dataset [19] taken from a study of the response of the human gut microbiome to a colonic cleanse. The original data contain 1651 leaves representing 1651 different taxa and we randomly selected a subtree with 50 leaves from the full tree.

We then obtained a Monte Carlo approximation to *E*(**MM**^*T*^) for this tree as described in Section 5.4. Also as before, we used a sequence of length 7 so that we could see the eigenvalues of the full range of possible values of *k* relative to the length of the sequence.

**Fig 7:**
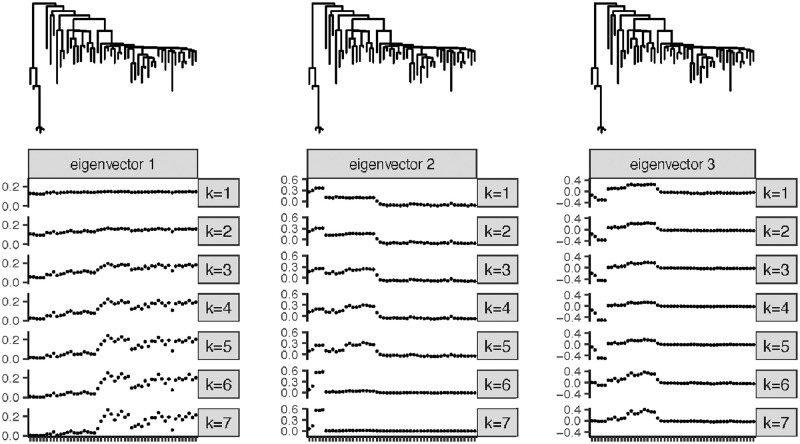
(Microbiome phylogeny) The first, second and third-largest eigenvector for different *k* values.

Fig 7 shows the first to third-largest eigenvectors of the simulated approximation of *E*(**MM**^*T*^) for the microbiome phylogeny using different values of *k*. The eigenvectors are qualitatively similar to those for the comb tree and the balanced binary tree. When *k* = 1, the first eigenvector is approximately constant, and when *k* = 7, the first eigenvector is approximately an indicator of the clade represented on the right-most side of the tree in the figure. This clade represents a group of closely-related taxa that are the most distinct from the rest of the taxa in the dataset, since they have a most-recent common ancestor that is relatively far from the root. The second eigenvector in each case is approximately an indicator of one of the clades. When *k* = 1, the second eigenvector is approximately an indicator of the clade of five taxa represented on the left of the tree. When *k* = 7, the second eigenvector is approximately an indicator of the subset of the three most-closely-related taxa in that clade. The third eigenvector is a contrast between clades for *k* = 1 and an indicator of one of the remaining clades for *k* = 7. In all cases, the intermediate eigenvectors are either intermediate between the *k* = 1 and *k* = 7 cases or have the same behavior as the *k* = 1 or *k* = 7 case. The phenomenon of the eigenvectors being either indicators of closely-related groups of taxa or contrasts between closely-related groups of taxa is consistent with the eigenvectors we saw in the more regular balanced binary and comb trees.

As with the comb tree and the balanced binary tree, the top eigenvalues have a much larger fraction of the weight when *k* is small than when *k* is large (see Fig 5, third panel). As before, this means that for small *k*, the top couple of eigenvector features are by far the most important, whereas for large *k* the subsequent eigenvectors are given relatively more weight.

### 3.6 *k*-mer-based distances and phylogenetic *β*-diversity measures are related

So far, we have shown that distances between *k*-mer spectra can be written as distances in a non-standard inner product space, where the matrix defining the inner product is **MM**^*T*^. We have also shown that *E*(**MM**^*T*^) tells us about the features used by *k*-mer-based distances, and that these features are related to the phylogeny. Our next task will be to show that one class of phylogenetic *β*-diversity measures, the MPQ distances (defined in Section 5.3), are similar to the EKS distance.

The MPQ distances are also generalized Euclidean distances, and the matrix **Q**_*r*_ (defined in Section 5.3, Eqs (3)–(6) plays the same role for the MPQ distances that **MM**^*T*^ does for the EKS distances. Therefore, just as we did with the EKS distances, we can understand the MPQ distances as weighted Euclidean distances that use features based on the eigenvectors of **Q**_*r*_ weighted by the eigenvalues of **Q**_*r*_. All the members of the MPQ family have the same set of eigenvectors. The difference is in the eigenvalues: the largest eigenvalues are much larger than the subsequent ones when *r* is close to 1, and the eigenvalues become more even as *r* goes to 0. The MPQ distance with *r* = 0 is the standard Euclidean distance between taxon abundance vectors.

We can show that the eigenvectors of **Q**_*r*_ are qualitatively similar to those of *E*(**MM**^*T*^), and in some cases are exactly the same. As seen in Section 4 of S1 Text and in Fig C in S1 Text, we see that the eigenvectors of **Q** are exactly the same as the eigenvectors of *E*(**MM**^*T*^) for the balanced binary tree. For the comb tree, especially for the largest eigenvector, we observe the S-shaped curve which looks similar to the largest eigenvector, and for the real tree, we see the same pattern of the eigenvectors being indicators of or contrasts between closely-related clades.

### 3.7 Computational complexity

As noted before (and as illustrated in Fig 1), the EKS distances and the MPQ distances are suited for different types of data. Given a marker gene sequencing dataset and a phylogenetic tree, it takes *O*(*p*^2^) to form the matrix **Q**_*r*_, since it is a function of the cophenetic distance matrix [20] and *O*(*np*^2^) to form the matrix of inner products. These inner products can often be used directly instead of the distances, but if distances are required they can be computed using an eigendecomposition of the inner product matrix, which will take time *O*(*n*^3^). For the EKS distances, once we have the *k*-mer spectra, and assuming K is the set of *k*-mers present across the samples, the computational complexity is $O(n^{2}|K|)$ to form the matrix of distances between *k*-mer spectra.

Therefore, assuming that we already have the phylogenetic tree or the *k*-mer spectra, computing the matrix of EKS distances will be faster if n|K| is small compared to *p*^2^. If *n* is on the same order as *p*, this will be true if we take a relatively small value of *k*. *k* = 5 gives us 1024 possible *k*-mers, *k* = 6 gives us 4096 *k*-mers (or half those numbers for canonical *k*-mers), both of which are on the order of the number of amplicon sequence variants (ASVs, see Glossary) present in stool samples. These values of *k* are also those that give us close matches with MPQ_1_, which is the MPQ distance that has been most commonly used in the microbiome literature. For larger values of *k*, the number of possible *k*-mers gets large very fast, but not quite as fast as it could because not all of the possible *k*-mers actually appear in the dataset. If *n* is very small relative to *p*, slightly larger values of *k* can lead to similar computational cost for the MPQ distances and the EKS distances.

Finally, notice that although the cost for computing the EKS distances is about the same as the cost for computing the MPQ distances when *k* is relatively small and gets more expensive than the MPQ distances for large *k*, if we wanted to compute the MPQ distance (or nearly any other phylogenetic *β*-diversity measure) on a shotgun metagenome sequencing dataset we would have to perform some costly pre-processing steps to get the information needed to compute the MPQ distances. In particular, we would have to assemble the sequences into contigs, get contig abundances, and estimate the phylogenetic relationships among the contigs to get the taxon abundances and phylogenetic tree required for these measures. Assembly is computationally costly, and it has the drawback of not using all of the reads. An alternate strategy could be to use Kraken 2/Bracken [21, 22] to estimate taxon abundances from the reads directly. The two drawbacks of this strategy are that Kraken 2/Bracken estimates taxonomy, which does not correspond exactly to the phylogeny that is required for the MPQ distances and that they are reference-based, meaning that the normal reference-free property of shotgun metagenome sequencing data is lost.

### 3.8 *k*-mer-based distances and phylogenetic *β*-diversity measures give similar results on real data

To show that our theoretical results carry through to real datasets, we compare EKS distances with different values of *k*, MPQ distances with different values of *r*, and the Euclidean distance on a real data set. The real data used in this section is a gut microbiome dataset [19] which focuses on the stability, resilience, and response to perturbation of the bacterial communities in the human gut. The dataset is a 16S rRNA sequencing dataset consisting of the abundances of 2611 amplicon sequence variants (ASVs). Each ASV is represented by a sequence of length 233, which we will use to compute the *k*-mer-based distances. Comparing the EKS distances to the MPQ distances is difficult because, as noted in the introduction, the MPQ distances require more information, and information of a different kind, than the *k*-mer-based distances. Normally MPQ distances would be computed using the sort of taxon abundance tables available in the 16S study we are considering here, while the EKS distances would be computed from shotgun sequencing data. However, we can compute EKS distances from the 16S sequences and use those as a basis for comparison. We will see that the EKS distances are almost identical to the MPQ distances.

After some filtering and other pre-processing (see Section 5.5 for details and Section 5 of S1 Text results of a parallel analysis with different pre-processing choices), we computed each type of distance between all pairs of samples. Therefore, for each type of distance, we have an *n* × *n* matrix giving the distances between each pair of samples, with the *i*, *j* element of the matrix corresponding to the distance between the *i*th and *j*th sample. We analyzed the resulting distances in two ways: first, we used DISTATIS [23] to visualize and quantify the similarity between every pair of *n* × *n* distance matrices. Second, we performed classical multi-dimensional scaling (CMDS) [24, 25] on each of the distance matrices to get a low-dimensional representation of the samples. These low-dimensional representations give a more qualitative understanding of the relationships among the distances.

**Fig 8:**
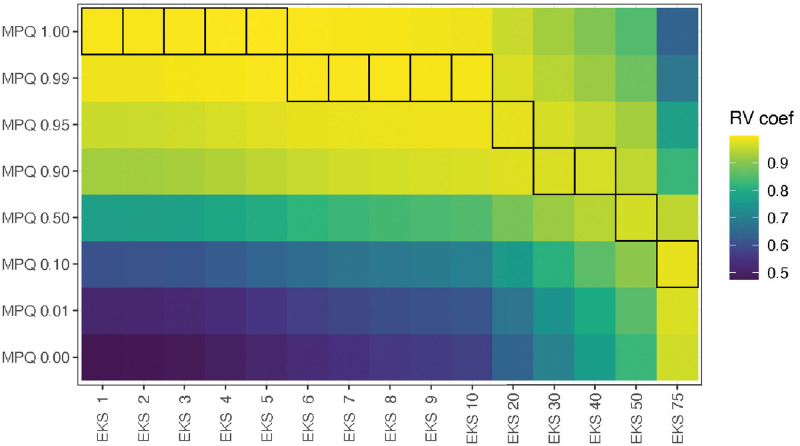
EKS distance matrices between the samples in the colon cleanout dataset are similar to the MPQ distance matrices. Each cell represents the RV coefficient between a pair of distance matrices, with yellow cells corresponding to large RV coefficients and purple corresponding to smaller RV coefficients. Outlined boxes indicate the maximum RV coefficient within a column.

**Fig 9:**
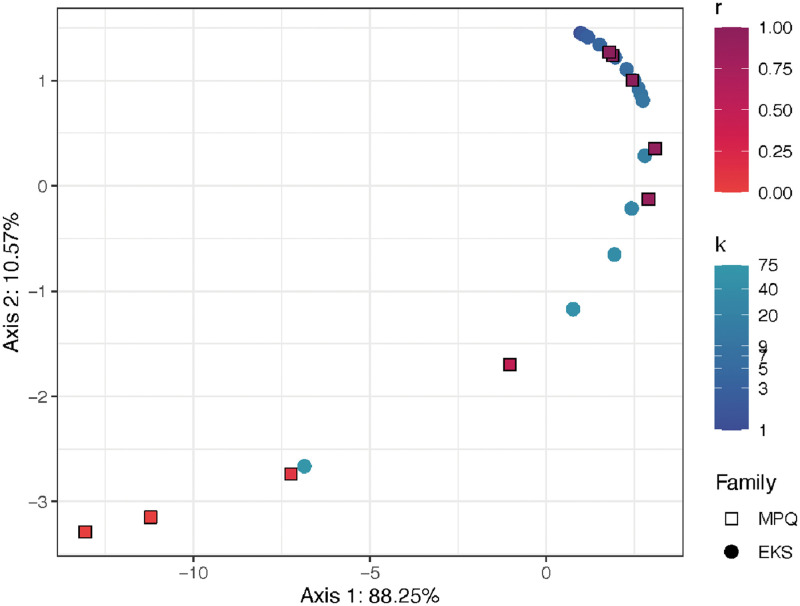
DISTATIS representation of the relationships among the EKS distances and MPQ distances. Each point represents a distance matrix describing the relationships among the samples in the colon cleanout dataset. Points corresponding to similar distance matrices are positioned close together in the plot. Squares represent EKS distance matrices, and circles represent MPQ distance matrices. Color represents the parameter (*k* or *r* for the EKS distances and MPQ distances). Darker colors represent larger values of *k*/smaller values of *r*, while lighter colors represent larger values of *r*/smaller values of *k*.

The results of DISTATIS are shown in Figs 8 and 9. Fig 8 shows the similarities between the EKS distances and the MPQ distances. DISTATIS measures similarities using the RV coefficient, which is a generalization of the correlation coefficient to multivariate data [26]. It takes values between 0 and 1, with larger values indicating a higher degree of similarity. Fig 8 shows us that for every EKS distance, there is a corresponding MPQ distance that matches it very closely. In the figure, for every EKS distance, the MPQ distance to which it is most similar is outlined in black. We see that the EKS distances with *k* from 1 to 5 are the most similar to the MPQ distance with *r* = 1, and the similarity is also very high at an absolute level, with RV coefficients very close to 1. The EKS distances with *k* from 5 to 10 are the most similar to the MPQ distance with a value of *r* equal to .99, *k* = 20 is most similar to MPQ distance with *r* = .95, and so on. The EKS distances with very large values of *k* become closer to the MPQ distance with small values of *r* or (equivalently) to the Euclidean distance between taxon abundance vectors. The EKS distance with *k* = 75 is closest to the MPQ distance with *r* = .1, but is also getting much more similar to the standard Euclidean distance. A table with the RV coefficients is included in the supplementary material (Table A in S1 Text).

The results from DISTATIS are visualized in a different way in Fig 9. In that plot, each point corresponds to a distance matrix, computed using either one of the MPQ distances or one of the EKS distances. The points are positioned in the space in such a way that small distances between points correspond to large RV coefficients between the corresponding distance matrices. Squares represent EKS distances and circles represent MPQ distances. We see that all of the distances, both EKS and MPQ, come very close to lying on a common curve, with location along the curve being governed by the parameter *k* for the EKS distances and the parameter *r* for the MPQ distances. The EKS distances with small values of *k* and the MPQ distances with *r* close to 1 lie to the upper right, and EKS distances with large values of *k* and MPQ distances with *r* close to 0 lie to the lower left. Finally, note that the fraction of variance explained by the principal plane is above 98%, indicating that nearly all the information encoded in the RV coefficient matrix is reflected in the plot.

**Fig 10:**
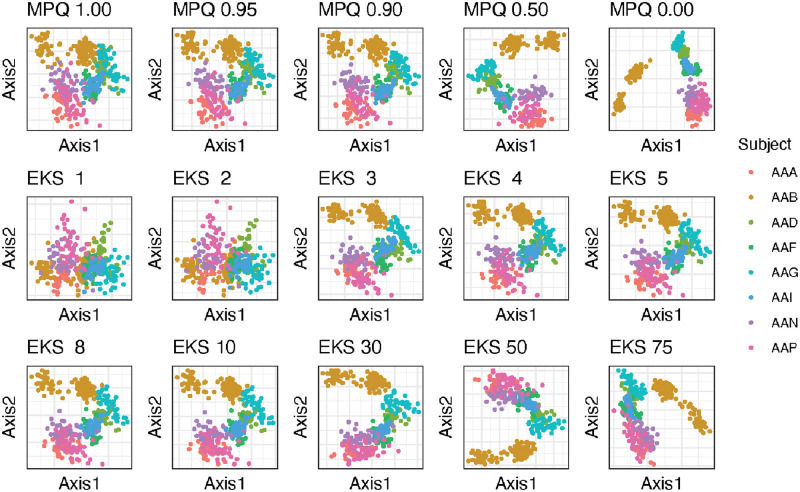
EKS distances with small values of *k* give representations of the colon cleanout samples that are similar to the MPQ distances with large values of *r*. Large values of *k* give representations of the samples that are similar to the MPQ distances with smaller values of *r*. For each type of distance, a distance matrix describing the relationships among the samples in the colon cleanout dataset was computed, CMDS was performed on the distance matrix, and the samples were plotted on the top two CMDS axes. Within each panel, each point corresponds to one sample in the colon cleanout dataset, and the color of the point represents the individual from whom the sample was taken.

Our last strategy for investigating the relationships among the distances under consideration here is to perform classical multi-dimensional scaling on each distance matrix and visualize the resulting representation of the samples. The results are given in Fig 10. We see that there are substantial differences in the representation of the samples depending on the value of *k* or *r* (for the EKS distances and MPQ distances, respectively), but that for a given EKS distance we can find an MPQ distance that gives a very similar embedding of the samples and vice versa. For both the EKS distances and the MPQ distances, distances at one end of the spectrum (large values of *k*/small values of *r*/the Euclidean distance) give representations of the samples in which the participants form distinct clusters. Distances on the other end of the spectrum (small values of *k*/large values of *r*) give representations of the samples with less distinct clusters by participant. Thus, the different members of both the EKS distances and the MPQ distances give substantially different distances between samples, but there is a very strong correspondence between the two classes. These results reinforce our theoretical results, showing that in real data, EKS distances mirror very closely the MPQ distances.

### 3.9 *k*-mer-based distances on a shotgun metagenome sequencing dataset

Finally, we show an example of using EKS distances on a recent study on the effect of non-nutritive sweeteners on the gut microbiome [27]. The data collected for the study were shotgun metagenome sequencing data. Since we do not have taxon abundances, we cannot compare the EKS distances to the MPQ distances. However, we can show how the *k*-mer-based distances vary across a range of values of *k*.

**Fig 11:**
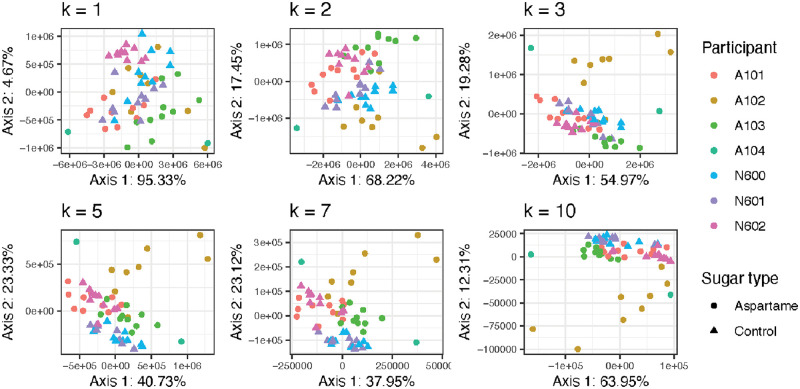
Visualization of the samples in the non-nutritive sweetener study using CMDS and the EKS distance for a range of values of *k*. For each value of *k*, the EKS distance was computed for every pair of samples. CMDS was run on the resulting distance matrices, giving an embedding of the samples into Euclidean space. The positions of the samples on the top two CMDS axes are shown for each value of *k*. Each point represents a sample, color represents the individual from whom the sample was taken, and shape represents the type of sweetener the participant was given (either aspartame or a control for the samples included here.).

We computed the *k*-mer spectra on a subset of the samples collected in this study using Jellyfish [28], computed EKS distances from the *k*-mer spectra, and performed CMDS on the resulting distances. The CMDS embeddings in the principal plane are given in Fig 11. The results are similar to, if less pronounced than, the colon cleanout dataset in the previous section. This dataset is again an experiment in which there are participant-specific and treatment-specific effects. At the small values of *k* the participant-specific effects (denoted by color in the figure) are less pronounced, and they become more apparent as *k* increases. Similarly, the treatment-specific effects are less pronounced overall than the subject-specific effects.

We can quantify the participant and treatment effects using the fraction of variance explained (*R*^2^) in a multivariate analysis of variance model (MANOVA). The *R*^2^ values for each value of *k* are given in Table 1. We see that the fraction of variance due to participant increases monotonically from *k* = 1 to *k* = 10 and is more than twice as large at the high end as at the low end. The fraction of variance due to the sugar type is lower overall and does not show the same monotone relationship with *k*: it peaks at *k* = 7, dropping back down with larger *k*. Overall, we see that, just as with the phylogenetically-informed *β*-diversity measures, the *k*-mer-based distances bring out different characteristics of the data as *k* changes.

**Table 1:** Fraction of variance explained by participant (left table) or sugar type (right table) for EKS distances with a range of values of *k*. Samples were embedded using CMDS and the EKS distance for the specified value of *k*, and MANOVA was used to compute the fraction of variance of the embedded points explained by participant or sugar type. The distance corresponding to the largest fraction of variance explained is highlighted in each table.

Participant		Sugar type	
*k*	*R* ^2^	*k*	*R* ^2^
1	0.059	1	0.018
2	0.086	2	0.040
3	0.101	3	0.048
5	0.121	5	0.061
7	0.131	**7**	**0.063**
**10**	**0.136**	10	0.046

## 4 Discussion

We showed that there is a strong relationship between a class of *k*-mer-based metagenome distances (the EKS distances) and a class of phylogenetically-informed *β*-diversity measures (the MPQ distances). Furthermore, we showed that both classes of distances can be interpreted as creating phylogenetically-informed features from the taxon abundances and computing a weighted Euclidean distance on those features. In the case of the balanced binary tree, those features are Felsenstein’s phylogenetically independent contrasts, and in other cases they tend to be either indicators of clades or contrasts between neighboring clades. In the distance computations, higher weights are given to features that are related to deep divisions in the tree, and the difference between EKS distances with different values of *k* and MPQ distances with different values of *r* is in how much more weight the deep-division features are given. Large *k* and small *r* correspond to less relative weight on the deep-division features, while small *k* and large *r* correspond to more relative weight on the deep-division features.

That the two kinds of distances should be related is implicit in some of the previous work with *k*-mers. For instance, taxonomic assignment methods are often based on the *k*-mer spectra for small values of *k*, generally on the order of 4–10 [30–35]. (Note that there is one outlier, [29], that gets best results for *k* on the order of 20 or 30.) Additionally, a preliminary step in metagenome assembly is sequence binning, and penta- or tetra-nucleotide spectra are often used for this task [36–38]. There have also been some limited empirical studies directed at examining the similarities between phylogenetically-informed *β*-diversity measures and *k*-mer-based metagenomic distances [39], but to our knowledge, this is the first time a formal relationship has been shown.

Our results here also shed some light on work by other groups: for instance, some machine learning methods have had success using *k*-mer features directly [40, 41]. Our results here suggest that the reason for their success is that the desired signal was in some phylogenetically-informed features which the *k*-mer representation implicitly picks out and emphasizes.

There are several limitations to the results in this paper. First of all, the matrix **MM**^*T*^ is random, and we have only looked at the eigenvectors and eigenvalues of its expectation, *E*(**MM**^*T*^). The expectation in question only takes into account variation in the random sequences that we obtain at the leaves of the tree when we assume a JC69 model of sequence evolution. The other source of variation that is present in metagenome studies is due to sampling *k*-mers: not every *k*-mer is sampled in these studies, and they are not sampled in exact proportion to the number of times they occur in the collection of genomes. It would also be of interest to quantify the variation that occurs in **MM**^*T*^ around its expected value, and to examine the distribution of the eigenvectors/eigenvalues of **MM**^*T*^ instead of examining the eigenvectors/eigenvalues of *E*(**MM**^*T*^). However, we have presented some evidence that the actual eigenvectors/eigenvalues of the random matrix **MM**^*T*^ are close to those of *E*(**MM**^*T*^) in the real data analysis presented in Section 3.8. The EKS distance (implicitly) uses **MM**^*T*^, not its expectation, and the fact that the EKS and MPQ distances led to very similar representations of the data suggests that the difference between **MM**^*T*^ and *E*(**MM**^*T*^) is not very large.

Some care should also be taken in using these results in situations that are very different from the model of sequence evolution that we assume. We would expect other Markov-type models of sequence evolution to give similar results, but if there is a substantial non-phylogenetic component in the sequences of the bacterial genomes, the EKS distances would reflect sequence similarity more than phylogenetic similarity. This could be the case, for instance, if there was a substantial amount of horizontal gene transfer.

Our work here is also useful for practitioners who would like to design phylogenetically-informed tests, regressions, or ordinations, but who only have *k*-mer data and lack taxon abundances and the phylogenetic tree. Our results show that working directly on the *k*-mer spectra is equivalent to using a phylogenetically-informed method. The results about small values of *k* corresponding to large weights on phylogenetically smooth features also gives guidance on the value of *k* to use: if the user believes the signal they are testing for is present in large clades or in taxa that are highly distinct from the rest of the tree, they would want to use a small value of *k*. On the other hand, if the user believes their signal is less phylogenetically smooth, perhaps scattered in several small clades, they would want to use a larger value of *k*. An interesting problem for future work would be to develop a procedure to adapt the value of *k* to a specific problem and dataset.

## 5 Methods

### 5.1 Weighted Euclidean distances

The weighted Euclidean distance is defined as follows: Suppose that **x**^*i*^ and **x**^*j*^ are vectors in $R^{p}$, and **w** is a vector in $R^{+}$ (all elements positive). We denote *m*th element of **x**^*i*^ as $x_{m}^{1}$, and similarly for **w**. Then the weighted Euclidean distance between **x**^*i*^ and **x**^*j*^ with weights **w** is given by


$$\begin{array}{c} d_{w}\left(x^{i},x^{j}\right)={\left[\sum_{m=1}^{p}w_{m}{\left(x_{m}^{1}-x_{m}^{2}\right)}^{2}\right]}^{1/2} \end{array}$$

### 5.2 Euclidean distances on *k*-mer spectra (EKS distances)

Suppose that we have *n* sequences or *n* metagenomes. Let $x^{i}\in R^{4^{k}}$ be the vector whose *m*th element, $x_{m}^{i}$ is the number of times we see the *j*th *k*-mer in the *i*th sequence or metagenome. The EKS_*k*_ distance between samples *i* and *j* is the Euclidean distance between **x**^*i*^ and **x**^*j*^, that is,

$$\begin{array}{cc} {\text{EKS}}_{k}\left(x^{i},x^{j}\right) & ={\left[\sum_{m=1}^{4^{k}}{\left(x_{m}^{i}-x_{m}^{j}\right)}^{2}\right]}^{1/2} \end{array}$$

### 5.3 Modulated phylogenetically-informed distances based on Rao’s quadratic entropy (MPQ distances)

Suppose that we have taxon abundances and a phylogenetic tree relating the taxa, as we would in an amplicon sequencing dataset. If $x^{i}\in R^{p}$ is a vector whose *j*th element gives the the abundance of taxon *j* in sample *i*, $Q\in R^{p\times p}$ is a matrix such that **Q**_*kl*_ is the amount of ancestral branch length shared by taxa *k* and *l*, and *r* is a number in [0, 1], the modulated phylogenetically-informed distance based on Rao’s quadratic entropy with parameter *r* between **x**^*i*^ and **x**^*j*^ is

$$\begin{array}{c} {\text{MPQ}}_{r}\left(x^{i},x^{j}\right)≔{\left(x^{i}-x^{j}\right)}^{T}Q_{r}\left(x^{i}-x^{j}\right) \end{array}$$ (2)

$$\begin{array}{cc} {\tilde{Q}}_{r} & ≔{\left(r^{-1}I_{p}+{\left(1-r\right)}^{-1}Q^{-1}\right)}^{-1},\;r\in \left(0,1\right) \end{array}$$ (3)

$$\begin{array}{cc} {\tilde{Q}}_{0} & ≔I_{p} \end{array}$$ (4)

$$\begin{array}{cc} {\tilde{Q}}_{1} & ≔Q \end{array}$$ (5)

$$\begin{array}{cc} Q_{r} & ≔{\tilde{Q}}_{r}/\text{trace}\left({\tilde{Q}}_{r}\right),\;r\in \left[0,1\right] \end{array}$$ (6)

This set of distances was described in [8] and was referred to in that paper as the “generalized DPCoA distances”. We have changed the name here because the distance is derived from Rao’s axiomatization of diversity and dissimilarity coefficients. The reasons for the components of the name are as follows:

- “Distance based on Rao’s quadratic entropy”: [9] described a measure of diversity that takes into account dissimilarities between taxa. He also described how to decompose his diversity measure to give a measure of distance between communities.
- “Phylogenetically-informed”: If we take the distances in the definition of Rao’s quadratic entropy to be the square root of the cophenetic distances between the taxa, we get a phylogenetically-informed distance between communities. [42] showed that this distance is the same as (**x**^*i*^ − **x**^*j*^)^*T*^ **Q**(**x**^*i*^ − **x**^*j*^), which is the MPQ distance with *r* = 1 and which is the distance in this family that has been used the most frequently.
- “Modulated”: The parameter *r* allows us to interpolate between the Euclidean distance (in the limit as *r* → 0) and the phylogenetic distance based on Rao’s quadratic entropy using the square roots of the cophenetic distances (in the limit as *r* → 1). We refer to the resulting distance with *r* ∈ (0, 1) as a *modulated* phylogenetically-informed distance because taking *r* to 0 gradually decreases the influence of the phylogenetic structure.

### 5.4 Monte Carlo Estimation of *E*(MM^*T*^)

To estimate *E*(**MM**^*T*^) for the balanced binary tree, comb tree, and the tree taken from a real dataset, we generated 10,000 random realizations of **MM**^*T*^ when the sequences of the taxa at the leaves of the tree came from the JC69 model. Specifically, we fixed the tree (the balanced binary tree, the comb tree or the real data tree), fixed a root sequence, and used pyvolve [43] 10,000 sets of *p* sequences, where *p* is the number of leaves in the tree. For the *r*th replicate, we let **M**^(*r*)^ be the *p* × 4^*k*^ matrix whose *i*, *j*th element is the number of times the *j*th *k*-mer was seen in the *i*th sequence. *E*(**MM**^*T*^) was then estimated as $\sum_{r=1}^{10000}M^{\left(r\right)}{\left(M^{\left(r\right)}\right)}^{T}/r$.

### 5.5 Distance computations and analysis of the colon cleanout dataset

The data were collected and processed as described in [19]. The data we used were stored as phyloseq objects, which we accessed from github.com/jfukuyama/DeepOrShallow. Taxa that were not seen with more than 3 counts in at least 20% of our samples were removed, giving a dateset with 419 (*n*) samples and 320 (*p*) taxa. Because of the skewness of the taxon abundances, it is standard to transform the taxon abundances. To check that the results are not unduly sensitive to the particular transformation used, we performed both a started log transformation, *x* ↦ log(1 + *x*), and a centered log-ratio transformation before centering the matrix. If **X** is the matrix with the raw data values and **X**_*clr*_ is the matrix containing the centered log-ratio values, we obtain the elements of **X**_*clr*_ as

$$\begin{array}{c} X_{clr_{\left(i,j\right)}}=log(\frac{X_{\left(i,j\right)}}{{\left(\prod_{j=1}^{p}X_{\left(i,j\right)}\right)}^{1/p}}). \end{array}$$

Because the MPQ and EKS distances are generalized Euclidean distances, and because of the equivalence between classical MDS and generalized PCA, the MDS embeddings for the MPQ distances were computed using the generalized PCA function in the adaptiveGPCA package. The data matrix was the same in each case, either the started log-transformed data or the centered log-ratio-transformed data. The matrix defining the inner product was either **Q**_*r*_ (MPQ distances), or **MM**^*T*^ (EKS distances). For the EKS distances, we notice that **MM**^*T*^ is the matrix of inner products between *k*-mer spectra, which is the spectrum kernel. We therefore used the stringdot function in the kernlab package [44, 45] to compute these values as it is a very efficient implementation.

DISTATIS was performed on the resulting set of distance matrices using the distatis function in the DistatisR package [46] in R. CMDS was performed with the cmds function in R. Plots were mode with the ggplot2 package [47].

### 5.6 Distance computations and analysis of the non-nutritive sweetener dataset

Shotgun metagenome sequencing data were downloaded from the European Nucleotide Archive. The samples were associated with project PRJEB47383, and a list of the accessions used is here given in Table C in S1 Text. For each sample, *k*-mer spectra were computed with Jellyfish [28]. Euclidean distances between the *k*-mer spectra were computed using a python script. Subsequent analysis of the distances using CMDS and MANOVA was performed in R using the cmds and manova functions. Plots were again generated using ggplot2. The set of bash, python, and R scripts used to go from a set of fasta files to the resulting figures is available at https://github.com/HongxuanZhai/k-mer-methods-and-phylogenetic-methods.

## Glossary

Some important terms:

- 16S rDNA sequencing study: A type of microbiome study in which DNA is extracted from a microbial community, a segment of the 16S rDNA gene is amplified and sequenced, and the resulting sequences, after either clustering or denoising, are as a proxy for the taxon abundances in the community.
- Amplicon sequencing study: A type DNA sequencing study in which a segment of a marker gene (usually the 16S rDNA gene for studies of bacterial genes and the internal transcribed spacer/ITS for studies of fungal communities) is amplified and sequenced. A 16S study is a type of amplicon seqeuncing study.
- Amplicon sequence variant (ASV): A proxy for bacterial species or strain in an amplicon sequencing study based on a denoised version of the raw, potentially noisy, sequences obtained from the sequencer.
- *β* diversity: A measure of the differences in taxon composition among communities.
- CMDS: Classical multi-dimensional scaling. A method that takes a set of distances between samples and creates a representation of the samples in Euclidean space so that the distances in the new space match the input distances.
- DISTATIS: Distance-based STATIS. STATIS is an abbreviation for the French *Structuration des Tableaux à Trois Indices de la Statistique*, and is a generalization of principal components analysis to three-dimensional arrays. Distance-based STATIS is a variant of STATIS that uses matrices of distances between samples instead of matrices containing variable measurements for the samples.
- *k*-mer spectrum: The number of times each *k*-mer is seen in a sequence or collection of sequences.
- MANOVA: Multivariate analysis of variance.
- MPQ distances: Modulated phylogenetically-informed distances based on Rao’s quadratic entropy. A set of phylogenetically-informed *β*-diversity measures or distances between microbial communities with a tuning parameter *r*. Designed for amplicon sequencing datasets for which one has a taxon abundance matrix and a phylogenetic tree.
- EKS distances: The Euclidean distances between *k*-mer spectra. A set of distances between metagenome samples.
- Shotgun metagenome sequencing study: A type of microbiome study in which DNA is extracted from a microbial community, broken into short fragments, and the fragments are sequenced.

## Supporting information

S1 Text Supplemental proofs, discussion, figures, and tables. **Fig A: The second, third and fourth-largest eigenvectors of the balanced binary tree.** **Fig B: The first three eigenvectors of the comb tree.** **Fig C: Eigenvectors of the Q matrix used in MPQ distances for different types of trees.** **Fig D: Embedding results for MPQ and EKS distances with different values of *r* and *k* on the CLR-transformed data.** **Fig E: Visualization of the RV coefficient matrix describing similarities of different MPQ ad EKS distances for CLR-transformed data.** Outlined box shows the MPQ distance that is the most similar to a given EKS distance. **Fig F: DISTATIS representation of the different MPQ and EKS distances using CLR-transformed data.** Squares represent EKS distances, circles represent MPQ distances. Darker colors correspond to small values of *r*/large values of *k*, and light colors correspond to large values of *r*/small values of *k*. **Table A: RV coefficient matrix describing similarities of MPQ distances with different values of *r* and EKS distances with different values of *k* using started log-transformed data.** **Table B: RV coefficient matrix describing similarities of MPQ distances with different values of *r* and EKS distances with different values of *k* for CLR-transformed data.** **Table C: Accessions and metadata for the samples used for the non-nutritive sweetener analysis**.
(PDF)
