# Supplementary material for: A convenient correspondence between k-mer-based metagenomic distances and phylogenetically-informed β-diversity measures
Source: PLoS Comput Biol. 2023 Jan 6;19(1):e1010821. doi: 10.1371/journal.pcbi.1010821 (PMC9879504; doi:10.1371/journal.pcbi.1010821)
Supplement: S1 Text — Fig A: The second, third and fourth-largest eigenvectors of the balanced binary tree. Fig B: The first three eigenvectors of the comb tree. Fig C: Eigenvectors of the Q matrix used in MPQ distances for different types of trees. Fig D: Embedding results for MPQ and EKS distances with different values of r and k on the CLR-transformed data. Fig E: Visualization of the RV coefficient matrix describing similarities of different MPQ ad EKS distances for CLR-transformed data. Outlined box shows the MPQ distance that is the most similar to a given EKS distance. Fig F: DISTATIS representation of the different MPQ and EKS distances using CLR-transformed data. Squares represent EKS distances, circles represent MPQ distances. Darker colors correspond to small values of r/large values of k, and light colors correspond to large values of r/small values of k. Table A: RV coefficient matrix describing similarities of MPQ distances with different values of r and EKS distances with different values of k using started log-transformed data. Table B: RV coefficient matrix describing similarities of MPQ distances with different values of r and EKS distances with different values of k for CLR-transformed data. Table C: Accessions and metadata for the samples used for the non-nutritive sweetener analysis. (PDF) [file pcbi.1010821.s001.pdf]

Supplemental Materials: *A convenient correspondence between k-mer-based metagenomic distances and phylogenetically-informed β-diversity measures*

Hongxuan Zhai and Julia Fukuyama

January 3, 2023

1 Blocked diagonal structure of $E(\mathbf{MM}^T)$ in balanced binary tree

Given a root sequence expression of length ℓ, there are $\ell - k + 1$ k-mers for $1 \le k \le \ell$. $\mathbf{M}$ can be decomposed directly with respect to those $\ell - k + 1$ k-mers,

$$\mathbf{M} = \sum_{i=1}^{\ell-k+1} \mathbf{M}_i,$$

where $\mathbf{M}_i$ is the counting matrix of size p by 4^k when we only look at the nucleotides in positions from i to $i + k - 1$ for each leaf sequence provided that $i + k - 1 \le \ell$.

With this, we have

$$\mathbf{MM}^T = \sum_{i=1}^{\ell-k+1} \mathbf{M}_i \sum_{j=1}^{\ell-k+1} \mathbf{M}_j^T.$$

The subscript i in $\mathbf{M}_i$ is indicating the starting position of a k-mer and given two subscripts i and j, we can find out if two $k$mers overlap and in which pattern they overlap. The idea is that suppose we do k-mer analysis and have two starting positions of k-mers i and j, we know that if $|i - j| \ge k$, then these two k-mers are "disjoint". And if $|i - j| = h$, $0 \le h \le k - 1$, these two k-mers share $k - h$ sites.

Then the $\mathbf{MM}^T$ can be written as

$$\begin{aligned}\mathbf{MM}^T = &\sum_{i=1}^{\ell-k+1} \sum_{|i-j|=0}^{\ell-k+1} \mathbf{M}_i\mathbf{M}_j^T + \sum_{i=1}^{\ell-k+1} \sum_{|i-j|=1}^{\ell-k+1} \mathbf{M}_i\mathbf{M}_j^T + \cdots \\ &+ \sum_{i=1}^{\ell-k+1} \sum_{|i-j|=k-1}^{\ell-k+1} \mathbf{M}_i\mathbf{M}_j^T + \sum_{i=1}^{\ell-k+1} \sum_{|i-j|\ge k}^{\ell-k+1} \mathbf{M}_i\mathbf{M}_j^T.\end{aligned}$$

Correspondingly, the expectation $E(\mathbf{MM}^T)$ can be expressed as

$$\begin{aligned}E(\mathbf{MM}^T) = &E\left(\sum_{i=1}^{\ell-k+1} \sum_{j:|i-j|=0}^{\ell-k+1} \mathbf{M}_i\mathbf{M}_j^T\right) + E\left(\sum_{i=1}^{\ell-k+1} \sum_{j:|i-j|=1}^{\ell-k+1} \mathbf{M}_i\mathbf{M}_j^T\right) + \cdots \\ &+ E\left(\sum_{i=1}^{\ell-k+1} \sum_{j:|i-j|=k-1}^{\ell-k+1} \mathbf{M}_i\mathbf{M}_j^T\right) + E\left(\sum_{i=1}^{\ell-k+1} \sum_{j:|i-j|\ge k}^{\ell-k+1} \mathbf{M}_i\mathbf{M}_j^T\right).\end{aligned}$$

Each M_i is filled with indicator random variables $m_{i,(n,g_a)} = \mathbb{1}(S_n^{(i,i+k-1)} = g_a)$, where $S_n^{(i,i+k-1)}$ is the random sequence expression from position i to $i + k - 1$ for leaf node n and g_a is some k-mer gene expression. To visualize,

$$\mathbf{M}_i = \begin{array}{c} \\ \\ \\ \\ \\ \\ \end{array}\begin{array}{c} \begin{matrix} g_1 & g_2 & \cdots & g_{4^k} \end{matrix} \\ \begin{pmatrix} m_{i,(1,g_1)} & m_{i,(1,g_2)} & \cdots & m_{i,(1,g_{4^k})} \\ m_{i,(2,g_1)} & m_{i,(2,g_2)} & \cdots & m_{i,(2,g_{4^k})} \\ \vdots & \vdots & \ddots & \vdots \\ m_{i,(p-1,g_1)} & m_{i,(p-1,g_2)} & \cdots & m_{i,(p-1,g_{4^k})} \\ m_{i,(p,g_1)} & m_{i,(p,g_2)} & \cdots & m_{i,(p,g_{4^k})} \end{pmatrix} \end{array} \begin{matrix} 1 \\ 2 \\ \vdots \\ p-1 \\ p \end{matrix}$$

1.1 Structure of $E(\sum_{i=1}^{\ell-k+1}\mathbf{M}_i\mathbf{M}_i^T)$

For each starting index i and leaf node u, the uth diagonal entry of $E(\mathbf{M}_i\mathbf{M}_i^T)$ is

$$\begin{aligned}E(\mathbf{M}_i\mathbf{M}_i^T)_{(u,u)} &= E\left(\sum_{a=1}^{4^k} m_{i,(u,g_a)}m_{i,(u,g_a)}|S_0^{(i,i+k-1)}\right)\\ &= \sum_{a=1}^{4^k} P\left(S_u^{(i,i+k-1)}=g_a|S_0^{(i,i+k-1)}\right)\\ &= 1.\end{aligned}$$

For a pair of different leaf nodes u and v and suppose their closest common ancestral node is c, we have

$$\begin{aligned}E(\mathbf{M}_i\mathbf{M}_i^T)_{(u,v)} &= E_k\left(\sum_{a=1}^{4^k} m_{i,(u,g_a)}m_{i,(v,g_a)}|S_0^{(i,i+k-1)}\right)\\ &= \sum_{a=1}^{4^k} P\left(S_u^{(i,i+k-1)}=g_a, S_v^{(i,i+k-1)}=g_a|S_0^{(i,i+k-1)}\right)\\ &= \sum_{a_1=1}^{4^k}\sum_{a_2=1}^{4^k} P\left(S_u^{(i,i+k-1)}=g_{a_1}|S_c^{(i,i+k-1)}=g_{a_2}\right)P\left(S_v^{(i,i+k-1)}=g_{a_1}|S_c^{(i,i+k-1)}=g_{a_2}\right)\\ &\qquad P\left(S_c^{(i,i+k-1)}=g_{a_2}|S_0^{(i,i+k-1)}\right)\\ &= \sum_{a_2=1}^{4^k} P\left(S_c^{(i,i+k-1)}=g_{a_2}|S_0^{(i,i+k-1)}\right)\left(\sum_{a_1=1}^{4^k} P\left(S_u^{(i,i+k-1)}=g_{a_1}|S_c^{(i,i+k-1)}=g_{a_2}\right)\right.\\ &\qquad\left. P\left(S_v^{(i,i+k-1)}=g_{a_1}|S_c^{(i,i+k-1)}=g_{a_2}\right)\right)\end{aligned}$$

Given any k-mer expression g_{a_2} of $S_c^{(i,i+k-1)}$, it is not hard to realize that when we sum over all possible k-mer expressions g_{a_1}, we have

$$\begin{aligned}&\sum_{a_1=1}^{4^k} P\left(S_u^{(i,i+k-1)}=g_{a_1}|S_c^{(i,i+k-1)}=g_{a_2}\right)P\left(S_v^{(i,i+k-1)}=g_{a_1}|S_c^{(i,i+k-1)}=g_{a_2}\right)\\ &\qquad= \sum_{j=0}^{k}\binom{k}{j}3^j p_0^{2(k-j)}(\frac{1-p_0}{3})^{2j} = (\frac{(1-p_0)^2}{3}+p_0^2)^k,\end{aligned}$$

where p_0 is the probability of no mutation based on the branch length from non-leaf node c to u and v in JC69 model [1].

1.2 Structure of $E(\sum_{i=1}^{\ell-k+1}\sum_{|i-j|\geq k}^{\ell-k+1}\mathbf{M}_i\mathbf{M}_j^T)$

Fix an starting index i and for any other starting position j such that $|i-j|\geq k$, the uth diagonal entry of $E(\mathbf{M}_i\mathbf{M}_j^T)$ is

$$\begin{aligned}E(\mathbf{M}_i\mathbf{M}_j^T)_{(u,u)} &= E\left(\sum_{a=1}^{4^k} m_{i,(u,g_a)}m_{j,(u,g_a)}|S_0^{(i,i+k-1)}, S_0^{(j,j+k-1)}\right)\\ &= \sum_{a=1}^{4^k} P\left(S_u^{(i,i+k-1)}=g_a|S_0^{(i,i+k-1)}\right)P\left(S_u^{(j,j+k-1)}=g_a|S_0^{(j,j+k-1)}\right).\end{aligned}$$

For a pair of different leaf nodes u and v, and w.l.o.g. assume starting index i is associated with u and j is associated with v. We have

$$E(\mathbf{M}_i\mathbf{M}_j^T)_{(u,v)} = E\left(\sum_{a=1}^{4^k} m_{i,(u,g_a)} m_{j,(v,g_a)} | S_0^{(i,i+k-1)}, S_0^{(j,j+k-1)}\right)$$
$$= \sum_{a=1}^{4^k} P\left(S_u^{(i,i+k-1)} = g_a | S_0^{(i,i+k-1)}\right) P\left(S_v^{(j,j+k-1)} = g_a | S_0^{(j,j+k-1)}\right).$$

Since u and v are both leaf nodes and the balanced binary tree is ultrametric, we have $E(\sum_{i=1}^{\ell-k+1}\sum_{|i-j|\geq k}^{\ell-k+1} \mathbf{M}_i\mathbf{M}_j^T)$ being a constant matrix.

1.3 Structure of $E(\sum_{i=1}^{\ell-k+1}\sum_{|i-j|=h}^{\ell-k+1} \mathbf{M}_i\mathbf{M}_j^T)$

Without loss of generality, fix an index i and j with $j-i=h$. Again, we examine the diagonals and off-diagonals in $E(\mathbf{M}_i\mathbf{M}_j^T)$. The uth diagonal entry is

$$E(\mathbf{M}_i\mathbf{M}_j^T)_{(u,u)} = E\left(\sum_{a=1}^{4^k} m_{i,(u,g_a)} m_{j,(u,g_a)} | S_0^{(i,j+k-1)}\right)$$
$$= \sum_{a=1}^{4^k} P\left(S_u^{(i,i+k-1)} = g_a, S_u^{(j,j+k-1)} = g_a | S_0^{(i,j+k-1)}\right).$$

We can see that, conditional on $S_0^{(i,j+k-1)}$, the diagonals will only be a function of the distance from the root to the tip u obtained from the phylogenetic tree. In the case when the tree is an ultrametric one, those distances will be the same and therefore all the diagonals are the same.

Consider a pair of two different leaf nodes u and v whose closest common ancestral node is c, the off-diagonal entries $E(\mathbf{M}_i\mathbf{M}_j^T)_{(u,v)}$ and $E(\mathbf{M}_i\mathbf{M}_j^T)_{(v,u)}$ are as follows,

$$E(\mathbf{M}_i\mathbf{M}_j^T)_{(u,v)} = E\left(\sum_{a=1}^{4^k} m_{i,(u,g_a)} m_{j,(v,g_a)} | S_0^{(i,j+k-1)}\right)$$
$$= \sum_{a=1}^{4^k} P\left(S_u^{(i,i+k-1)} = g_a, S_v^{(j,j+k-1)} = g_a | S_0^{(i,j+k-1)}\right)$$
$$= \sum_{a_1=1}^{4^k}\sum_{a_2=1}^{4^{(k+h)}} P\left(S_u^{(i,i+k-1)} = g_{a_1} | S_c^{(i,j+k-1)} = g_{a_2}\right) P\left(S_v^{(j,j+k-1)} = g_{a_1} | S_c^{(i,j+k-1)} = g_{a_2}\right)$$
$$P\left(S_c^{(i,j+k-1)} = g_{a_2} | S_0^{(i,j+k-1)}\right)$$
$$= \sum_{a_2=1}^{4^{(k+h)}} P\left(S_c^{(i,j+k-1)} = g_{a_2} | S_0^{(i,j+k-1)}\right) \left(\sum_{a_1=1}^{4^k} P\left(S_u^{(i,i+k-1)} = g_{a_1} | S_c^{(i,j+k-1)} = g_{a_2}\right)\right.$$
$$\left. P\left(S_v^{(j,j+k-1)} = g_{a_1} | S_c^{(i,j+k-1)} = g_{a_2}\right)\right)$$
$$= \sum_{a_2=1}^{4^{(k+h)}} P\left(S_c^{(i,j+k-1)} = g_{a_2} | S_0^{(i,j+k-1)}\right) \left(\sum_{a_1=1}^{4^k} P\left(S_u^{(i,i+k-1)} = g_{a_1} | S_c^{(i,i+k-1)} = g_{a_2}^{(1,k)}\right)\right.$$
$$\left. P\left(S_v^{(j,j+k-1)} = g_{a_1} | S_c^{(j,j+k-1)} = g_{a_2}^{(h+1,k+h)}\right)\right),$$

and

$$E(\mathbf{M}_i\mathbf{M}_j^T)_{(v,u)} = E\left(\sum_{a=1}^{4^k} m_{i,(v,g_a)}m_{j,(u,g_a)}|S_0^{(i,j+k-1)}\right)$$
$$= \sum_{a_2=1}^{4^{(k+h)}} P\left(S_c^{(i,j+k-1)} = g_{a_2}|S_0^{(i,j+k-1)}\right)\left(\sum_{a_1=1}^{4^k} P\left(S_v^{(i,i+k-1)} = g_{a_1}|S_c^{(i,i+k-1)} = g_{a_2}^{(1,k)}\right)\right.$$
$$\left. P\left(S_u^{(j,j+k-1)} = g_{a_1}|S_c^{(j,j+k-1)} = g_{a_2}^{(h+1,k+h)}\right)\right).$$

where g_{a_2} is the generic expression for any $(k+h)$-mer and superscripts denote positions. With the complete binary tree and conditional on the information of the corresponding ancestral subsequence, each term will be only a function of the distance between tip u and v so that for each pair of i and j, $E(\mathbf{M}_i\mathbf{M}_j^T)$ is symmetric. Therefore, $E(\sum_{i=1}^{\ell-k+1}\sum_{|i-j|=h}^{\ell-k+1}\mathbf{M}_i\mathbf{M}_j^T)$ is symmetric.

Now consider a triplet of different leaf nodes u, v_1 and v_2 and denote the most recent common ancestral node of u and v_1 being c_1 and c_2 for u and v_2. Two quantities that we want to examine are

$$E(\mathbf{M}_i\mathbf{M}_j^T)_{(u,v_1)} = \sum_{a_2=1}^{4^{(k+h)}} P\left(S_{c_1}^{(i,j+k-1)} = g_{a_2}|S_0^{(i,j+k-1)}\right)\sum_{a_1=1}^{4^k} P\left(S_u^{(i,i+k-1)} = g_{a_1}, S_{v_1}^{(j,j+k-1)} = g_{a_1}|S_{c_1}^{(i,j+k-1)} = g_{a_2}\right)$$
$$= \sum_{a_2=1}^{4^{(k+h)}} P\left(S_{c_1}^{(i,j+k-1)} = g_{a_2}|S_0^{(i,j+k-1)}\right)\left(\sum_{a_1=1}^{4^k} P\left(S_u^{(i,i+k-1)} = g_{a_1}|S_{c_1}^{(i,j+k-1)} = g_{a_2}\right)\right.$$
$$\left. P\left(S_{v_1}^{(j,j+k-1)} = g_{a_1}|S_{c_1}^{(i,j+k-1)} = g_{a_2}\right)\right)$$
$$= \sum_{a_2=1}^{4^{(k+h)}} P\left(S_{c_1}^{(i,j+k-1)} = g_{a_2}|S_0^{(i,j+k-1)}\right)\left(\sum_{a_1=1}^{4^k} P\left(S_u^{(i,i+k-1)} = g_{a_1}|S_{c_1}^{(i,i+k-1)} = g_{a_2}^{(1,k)}\right)\right.$$
$$\left. P\left(S_{v_1}^{(j,j+k-1)} = g_{a_1}|S_{c_1}^{(j,j+k-1)} = g_{a_2}^{(h+1,k+h)}\right)\right),$$

and

$$E(\mathbf{M}_i\mathbf{M}_j^T)_{(u,v_2)} = \sum_{a_2=1}^{4^{(k+h)}} P\left(S_{c_2}^{(i,j+k-1)} = g_{a_2}|S_0^{(i,j+k-1)}\right)\left(\sum_{a_1=1}^{4^k} P\left(S_u^{(i,i+k-1)} = g_{a_1}|S_{c_2}^{(i,i+k-1)} = g_{a_2}^{(1,k)}\right)\right.$$
$$\left. P\left(S_{v_1}^{(j,j+k-1)} = g_{a_1}|S_{c_2}^{(j,j+k-1)} = g_{a_2}^{(h+1,k+h)}\right)\right).$$

Recall that with the ultrametric balanced binary phylogenetic tree, given a pair of tips u and v and their most recent common ancestral node c, we have

$$\begin{aligned} d(r,u) &= d(r,v) \\ &= d(c,v) + d(r,c) \\ &= d(c,u) + d(r,c) \\ &= \frac{1}{2}d(u,v) + d(r,c), \end{aligned}$$

and

$$\begin{aligned} d(r,c) &= \frac{1}{2}d(u,v) - d(r,u) \\ &= \frac{1}{2}d(u,v) - d(r,v). \end{aligned}$$

where $d(\cdot,\cdot)$ is the distance function on the tree and r is the root of the tree.

For each g_{a_2} and g_{a_1}, $P\left(S_{c_1}^{(i,j+k-1)} = e_{a_2}|S_0^{(i,j+k-1)}\right)$ is a function of $d(r, c_1)$ as well as a function of $d(u, v_1)$. $P\left(S_u^{(i,i+k-1)} = g_{a_1}|S_{c_1}^{(i,i+k-1)} = g_{a_2}^{(1,k)}\right)$ and $P\left(S_{v_1}^{(j,j+k-1)} = g_{a_1}|S_{c_1}^{(j,j+k-1)} = g_{a_2}^{(h+1,k+h)}\right)$ are both functions of $d(u, v_1)$. Similarly, $P\left(S_{c_2}^{(i,j+k-1)} = g_{a_2}|S_0^{(i,j+k-1)}\right)$, $P\left(S_u^{(i,i+k-1)} = g_{a_1}|S_{c_2}^{(i,i+k-1)} = g_{a_2}^{(1,k)}\right)$ and $P\left(S_{v_2}^{(j,j+k-1)} = g_{a_1}|S_{c_2}^{(j,j+k-1)} = g_{a_2}^{(h+1,k+h)}\right)$ are functions of $d(u, v_2)$. From this perspective, we conclude that the off-diagonals $E(\mathbf{M}_i\mathbf{M}_j^T)_{(u,v_1)}$ and $E(\mathbf{M}_i\mathbf{M}_j^T)_{(u,v_2)}$ only differ when $d(u, v_1) \neq d(u, v_2)$, that is when the tips v_1 and v_2 do not share the same most recent common ancestral node with respect to u. Therefore, by examing the distance between tips on the complete binary tree, we have that $E(\sum_{i=1}^{\ell-k+1}\sum_{|i-j|=h}^{\ell-k+1}\mathbf{M}_i\mathbf{M}_j^T)$ is in the blocked diagonal form. Then, we established the fact that with JC69 model and balanced binary phylogeny, $E(\mathbf{M}\mathbf{M}^T)$ is in the blocked diagonal form.

2 Eigenstructure of $E(\mathrm{MM}^T)$ in balanced binary tree

Assume the balanced binary tree has depth d (root r is in depth 1), then the form of the blocked diagonal matrix $\mathbf{D}$, in which the entries are denoted as μ's can be expressed as follows,

$$\mathbf{D} = \left(\begin{array}{c|c} \mathbf{D}_1 & \mu_{d-1}\mathbf{J}_{2^{d-2}} \\ \hline \mu_{d-1}\mathbf{J}_{2^{d-2}} & \mathbf{D}_1 \end{array}\right),$$

in which $\mathbf{D}_1$ is also a blocked diagonal matrix in form of

$$\left(\begin{array}{c|c} \mathbf{D}_1^{'} & \mu_{d-2}\mathbf{J}_{2^{d-3}} \\ \hline \mu_{d-2}\mathbf{J}_{2^{d-3}} & \mathbf{D}_1^{'} \end{array}\right),$$

and $\mathbf{J}_n$ is an all-ones square matrix of size n.

2.1 Eigenvalues of D

$\mathbf{D}$ is a non-negative matrix and each row of $\mathbf{D}$ sums up to the same number. By Perron–Frobenius theorem, we immediately have the leading eigenvalue λ_1 is the row sum. By the fact that determinant of $\mathbf{D}$ is $det(\mathbf{D}_1 - \mu_{d-1}\mathbf{J}_{2^{d-2}})det(\mathbf{D}_1 + \mu_{d-1}\mathbf{J}_{2^{d-2}})$ and $\mathbf{D}_1$ is also in the same matrix form (with a smaller size) and $\mu_{d-1}\mathbf{J}_{2^{d-2}}$ is a constant matrix, we conclude that both $\mathbf{D}_1 - \mu_{d-1}\mathbf{J}_{2^{d-2}}$ and $\mathbf{D}_1 + \mu_{d-1}\mathbf{J}_{2^{d-2}}$ are also in the same block form as $\mathbf{D}_1$. Therefore, the determinant of $\mathbf{D}$ can be found in a recursive manner.

The characteristic polynomial $p_d(\lambda)$of $\mathbf{D}$ is

$$\begin{aligned}
p_d(\lambda) = &\left((\mu_0 + \sum_{i=2}^{d-1} 2^{i-2}\mu_i - \lambda)^2 - (\mu_1 + \sum_{i=2}^{d-1} 2^{i-2}\mu_i)^2\right) \\
&\times \left((\mu_0 + \sum_{i=2}^{d-2} 2^{i-2}\mu_i - 2^{d-3}\mu_{d-1} - \lambda)^2 - (\mu_1 + \sum_{i=2}^{d-2} 2^{i-2}\mu_i - 2^{d-3}\mu_{d-1})^2\right) \\
&\times \left((\mu_0 + \sum_{i=2}^{d-3} 2^{i-2}\mu_i - 2^{d-4}\mu_{d-2} - \lambda)^2 - (\mu_1 + \sum_{i=2}^{d-3} 2^{i-2}\mu_i - 2^{d-4}\mu_{d-2})^2\right)^2 \times \cdots \\
&\times \left((\mu_0 + \sum_{i=2}^{d-j} 2^{i-2}\mu_i - 2^{d-(j+1)}\mu_{d-(j-1)} - \lambda)^2 - (\mu_1 + \sum_{i=2}^{d-j} 2^{i-2}\mu_i - 2^{d-(j+1)}\mu_{d-(j-1)})^2\right)^{2^{j-2}} \times \cdots \\
&\times \left((\mu_0 + \sum_{i=2}^{d-(d-1)} 2^{i-2}\mu_i - 2^{d-d}\mu_{d-(d-2)} - \lambda)^2 - (\mu_1 + \sum_{i=2}^{d-(d-1)} 2^{i-2}\mu_i - 2^{d-d}\mu_{d-(d-2)})^2\right)^{2^{d-3}},
\end{aligned}$$

through which the all the eigenvalues and their multiplicities can be identified.

2.2 Eigenspace of D

Based on the block structure of $\mathbf{D} \in \mathbb{R}^{2^j \times 2^j}$, where 2^j is the total number of leaves in the balanced binary tree, we show here how to write $\mathbf{D}$ in terms of a linear combination of $\mathbf{D}^{ij}$. $\mathbf{D}^{ij}$ is a $2^j \times 2^j$ matrix with $2^i \times 2^i$ blocks of 1's on the diagonals.

Let $\mathbf{c}^{ijk}$ denote a vector of length 2^j with a block of 2^{i+1} non-zero elements, with the first 2^i of the non-zero elements equal to 1 and the second block of 2^i non-zero elements equal to -1. k can be considered as the index for such vectors and for fixed values of i and j, we have 2^{j-i-1} possibilities for k.

Notice that if we have $\mathbf{c}^{i_2jk}$ is an eigenvector of $\mathbf{D}^{i_1j}$ if $i_2 \geq i_1$. Furthermore, the $\mathbf{c}^{ijk}$'s form an orthogonal basis for $\mathbb{R}^{2^j}$. If $i < j-1$, we can write

$$\mathbf{D}^{ij} = \frac{1}{2}\left[\sum_{k'} \mathbf{c}^{ijk'}(\mathbf{c}^{ijk'})^T + \mathbf{D}^{(i+1)j}\right]$$

If $i = j-1$, we have

$$\mathbf{D}^{ij} = \frac{1}{2}(\mathbf{c}^{ij0}(\mathbf{c}^{ij0})^T + \mathbf{c}^{jj0}(\mathbf{c}^{jj0})^T)$$

and if $i = j$, we have

$$\mathbf{D}^{ij} = \mathbf{c}^{jj0}(\mathbf{c}^{jj0})^T$$

This means that for any $i < j$, we have

$$\mathbf{D}^{ij} = \sum_{\ell=0}^{j-i-1} \frac{1}{2^{\ell+1}} \sum_{k'} \mathbf{c}^{(i+\ell)jk'}(\mathbf{c}^{(i+\ell)jk'})^T + \frac{1}{2^{j-i}}\mathbf{c}^{jj0}(\mathbf{c}^{jj0})^T$$

Finally, if the number of leaves on our tree is 2^j, notice that we can write $E(\mathbf{M}\mathbf{M}^T)$ as

$$\begin{aligned} &E(\mathbf{M}\mathbf{M}^T) \\ &= \sum_{i=0}^{j-1} (\mu_i - \mu_{i+1})\,\mathbf{D}^{ij} + \mu_j \mathbf{D}^{jj} \\ &= \sum_{i=0}^{j-1} (\mu_i - \mu_{i+1}) \left[\sum_{\ell=0}^{j-i-1} \frac{1}{2^{\ell+1}} \sum_{k'} \mathbf{c}^{(i+\ell)jk'}(\mathbf{c}^{(i+\ell)jk'})^T + \frac{1}{2^{j-i}}\mathbf{c}^{jj0}(\mathbf{c}^{jj0})^T\right] + \mu_j \mathbf{c}^{jj0}(\mathbf{c}^{jj0})^T \end{aligned}$$

We can get the eigenvalues in two pieces. The eigenvalue corresponding to $\mathbf{c}^{jj0}$ will be

$$\lambda_j = \|\mathbf{c}^{jj0}\|^2 \left(\mu_j + \sum_{i=0}^{j-1}(\mu_i - \mu_{i+1})\frac{1}{2^{j-i}}\right) = 2^j \mu_j + \sum_{i=0}^{j-1} 2^i(\mu_i - \mu_{i+1})$$

This tells us that the eigenvalue corresponding to the eigenvector $\mathbf{c}^{ijk}$ is

$$\lambda_i = \|\mathbf{c}^{ijk}\|^2 \sum_{i',\ell:i'+\ell=i} \frac{\mu_{i'} - \mu_{i'+1}}{2^{\ell+1}} = 2^{i+1} \sum_{i'=0}^{i} \frac{\mu_{i'} - \mu_{i'+1}}{2^{i-i'+1}} = \sum_{i'=0}^{i} 2^{i'}(\mu_{i'} - \mu_{i'+1})$$

3 Eigenstructure of $E(\mathrm{MM}^T)$ for trees with 128 tips

To better demonstrate our findings of the eigenvectors in the binary tree and comb tree, we repeat the analysis with trees with more leaves.

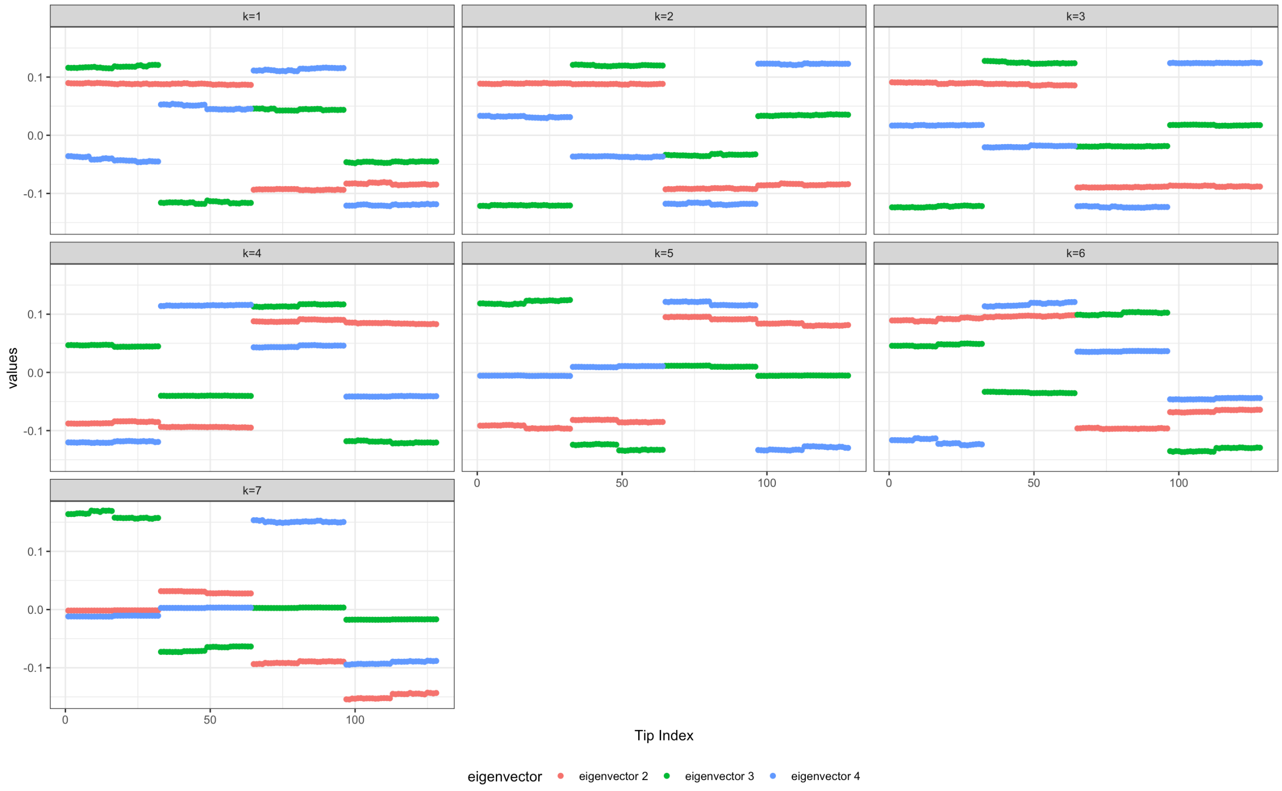

Fig A: The second, third and fourth-largest eigenvectors of the balanced binary tree.

The features defined by eigenvectors for a balanced binary tree with 128 tips are still in the same pattern as what we find in the paper: features with progressively smaller eigenvalues measure the difference in abundance between successively smaller clades and their sisters.

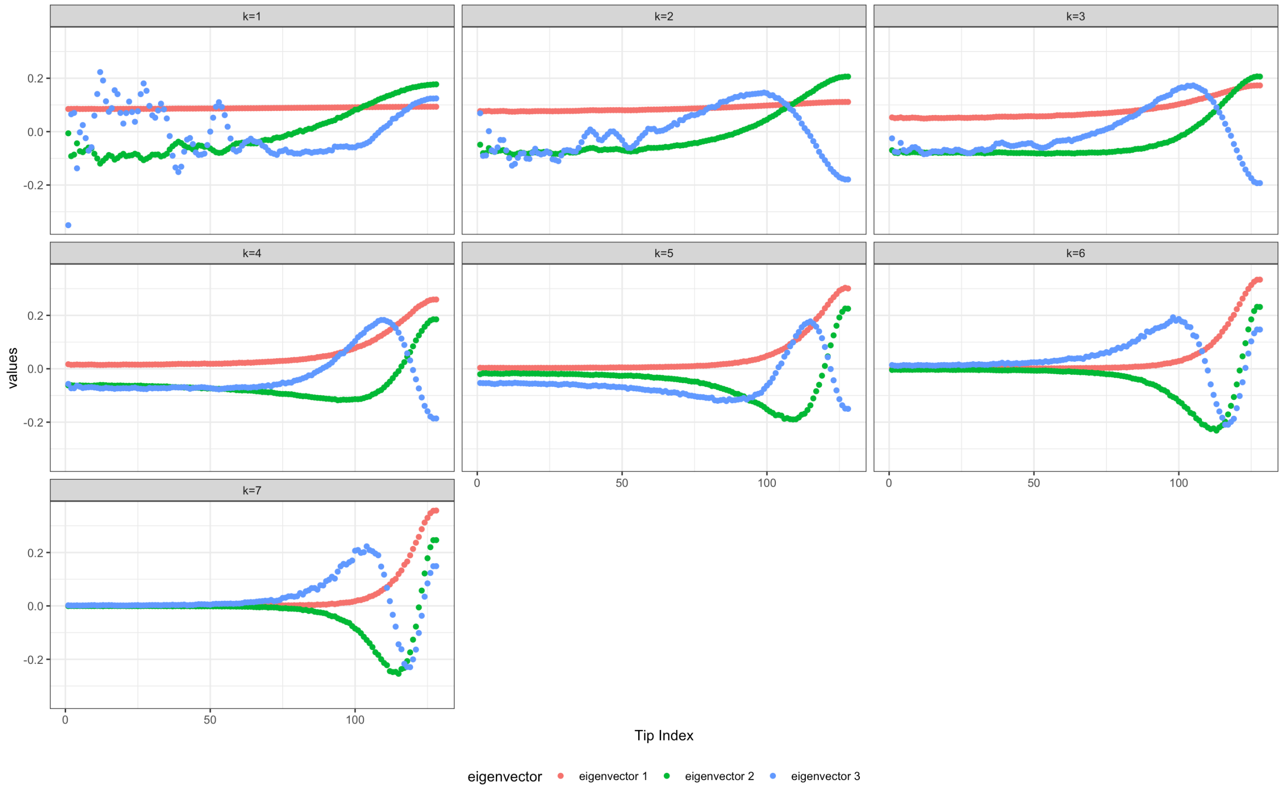

Fig B: The first three eigenvectors of the comb tree.

It is clear that the first-largest eigenvectors (features) behave the same as the features in Figure 6 in the paper and they have the same interpretations. With more leaves on the comb tree, it is more apparent that features in the comb tree are combinations of features that are contrasts between close sister clades and contrasts between most distinct taxa. With small k between 1 and 3, the third-largest eigenvectors can be considered as both contrast between the taxa in the left-hand side and contrast between two close sister clades in the right-hand side. When k is between 4 to 7, the pattern of being a contrast between one part of the tree and another is more evident.

4 Eigenstructure of Q in MPQ distances

The relationship between EKS distances and MPQ distances can be established by investigating the eigenstructure of the inner product matrix $\mathbf{Q}_r$ used in the MPQ distances.

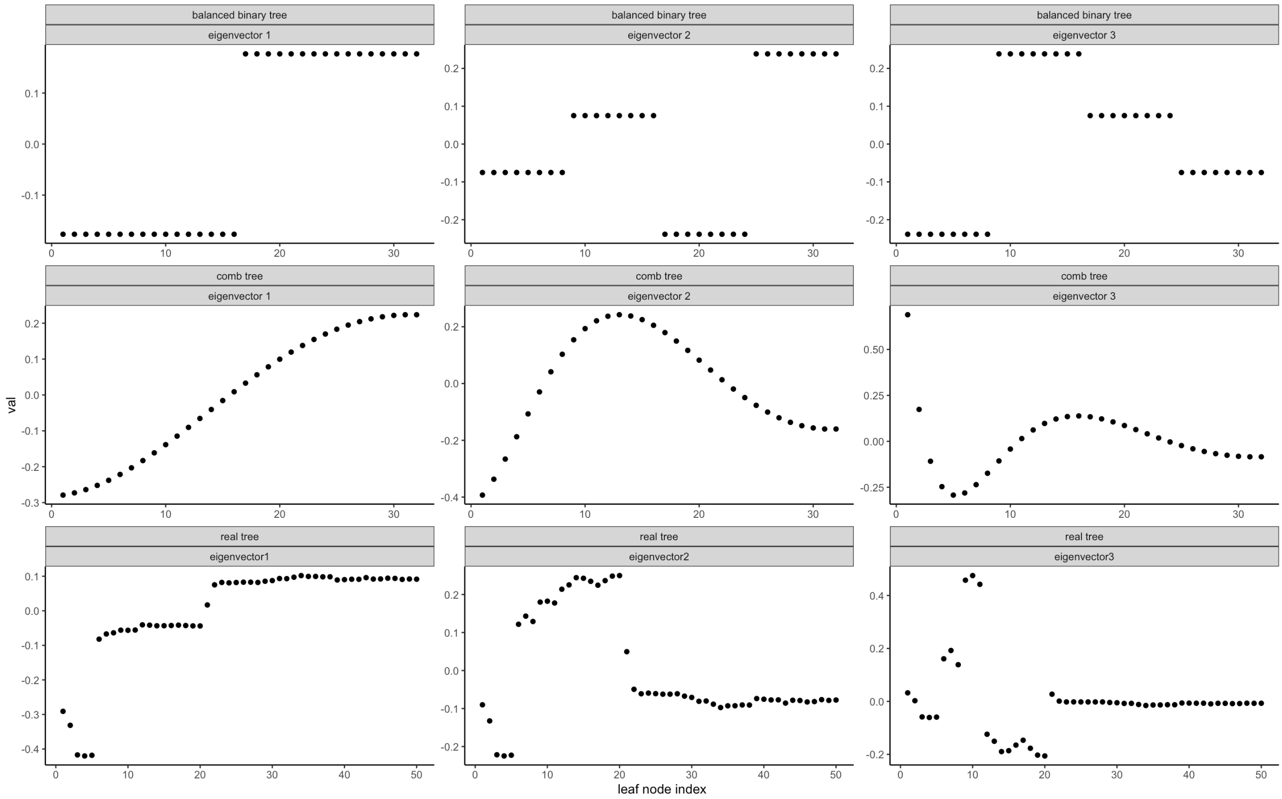

Fig C: Eigenvectors of the $\mathbf{Q}$ matrix used in MPQ distances for different types of trees.

The eigenstructures of $\mathbf{Q}$ are similar to those in $E(\mathbf{MM}^T)$ with the balanced binary tree. In other settings, although the eigenvectors don't look exactly the same, those eigenvectors are either trying to build contrast between close-related clades or contrast between more distinct taxa or achieve both.

	$r=0$	$r=.50$	$r=.90$	$r=.95$	$r=1.00$	$k=1$	$k=2$	$k=3$	$k=4$	$k=5$	$k=8$	$k=10$	$k=30$	$k=50$	$k=75$
$r=0$	1.00	0.89	0.71	0.65	0.51	0.48	0.48	0.48	0.49	0.51	0.56	0.57	0.71	0.83	0.96
$r=.50$	0.89	1.00	0.94	0.90	0.80	0.77	0.77	0.77	0.79	0.80	0.84	0.85	0.92	0.97	0.95
$r=.90$	0.71	0.94	1.00	0.99	0.95	0.93	0.93	0.93	0.94	0.95	0.97	0.97	0.97	0.95	0.82
$r=.95$	0.65	0.90	0.99	1.00	0.97	0.96	0.96	0.96	0.97	0.98	0.99	0.99	0.97	0.93	0.77
$r=1.00$	0.51	0.80	0.95	0.97	1.00	1.00	1.00	1.00	1.00	1.00	0.99	0.99	0.93	0.85	0.64
$k=1$	0.48	0.77	0.93	0.96	1.00	1.00	1.00	1.00	1.00	1.00	0.98	0.97	0.90	0.82	0.61
$k=2$	0.48	0.77	0.93	0.96	1.00	1.00	1.00	1.00	1.00	1.00	0.98	0.97	0.90	0.82	0.61
$k=3$	0.48	0.77	0.93	0.96	1.00	1.00	1.00	1.00	1.00	1.00	0.98	0.98	0.91	0.83	0.62
$k=4$	0.49	0.79	0.94	0.97	1.00	1.00	1.00	1.00	1.00	1.00	0.99	0.98	0.92	0.84	0.63
$k=5$	0.51	0.80	0.95	0.98	1.00	1.00	1.00	1.00	1.00	1.00	0.99	0.99	0.93	0.86	0.65
$k=8$	0.56	0.84	0.97	0.99	0.99	0.98	0.98	0.98	0.99	0.99	1.00	1.00	0.96	0.89	0.70
$k=10$	0.57	0.85	0.97	0.99	0.99	0.97	0.97	0.98	0.98	0.99	1.00	1.00	0.97	0.91	0.71
$k=30$	0.71	0.92	0.97	0.97	0.93	0.90	0.90	0.91	0.92	0.93	0.96	0.97	1.00	0.98	0.84
$k=50$	0.83	0.97	0.95	0.93	0.85	0.82	0.82	0.83	0.84	0.86	0.89	0.91	0.98	1.00	0.93
$k=75$	0.96	0.95	0.82	0.77	0.64	0.61	0.61	0.62	0.63	0.65	0.70	0.71	0.84	0.93	1.00

Table A: RV coefficient matrix describing similarities of MPQ distances with different values of r and EKS distances with different values of k using started log-transformed data.

	$r=0$	$r=.50$	$r=.90$	$r=.95$	$r=1.00$	$k=3$	$k=4$	$k=5$	$k=10$	$k=20$	$k=30$	$k=40$	$k=50$	$k=75$	$k=150$
$r=0$	1.00	0.95	0.87	0.85	0.81	0.83	0.85	0.86	0.87	0.91	0.94	0.95	0.97	0.98	1.00
$r=.50$	0.95	1.00	0.97	0.96	0.93	0.91	0.94	0.95	0.95	0.96	0.97	0.97	0.98	0.97	0.96
$r=.90$	0.87	0.97	1.00	1.00	0.99	0.92	0.97	0.97	0.97	0.96	0.95	0.94	0.93	0.91	0.88
$r=.95$	0.85	0.96	1.00	1.00	0.99	0.92	0.97	0.97	0.97	0.95	0.94	0.92	0.91	0.89	0.86
$r=1.00$	0.81	0.93	0.99	0.99	1.00	0.91	0.96	0.96	0.96	0.93	0.91	0.89	0.88	0.86	0.82
$k=3$	0.83	0.91	0.92	0.92	0.91	1.00	0.97	0.95	0.94	0.93	0.92	0.91	0.90	0.88	0.84
$k=4$	0.85	0.94	0.97	0.97	0.96	0.97	1.00	0.99	0.99	0.97	0.96	0.94	0.93	0.90	0.86
$k=5$	0.86	0.95	0.97	0.97	0.96	0.95	0.99	1.00	0.99	0.98	0.97	0.95	0.94	0.91	0.88
$k=10$	0.87	0.95	0.97	0.97	0.96	0.94	0.99	0.99	1.00	0.99	0.98	0.96	0.95	0.93	0.89
$k=20$	0.91	0.96	0.96	0.95	0.93	0.93	0.97	0.98	0.99	1.00	1.00	0.99	0.98	0.96	0.92
$k=30$	0.94	0.97	0.95	0.94	0.91	0.92	0.96	0.97	0.98	1.00	1.00	1.00	0.99	0.98	0.95
$k=40$	0.95	0.97	0.94	0.92	0.89	0.91	0.94	0.95	0.96	0.99	1.00	1.00	1.00	0.99	0.96
$k=50$	0.97	0.98	0.93	0.91	0.88	0.90	0.93	0.94	0.95	0.98	0.99	1.00	1.00	0.99	0.97
$k=75$	0.98	0.97	0.91	0.89	0.86	0.88	0.90	0.91	0.93	0.96	0.98	0.99	0.99	1.00	0.98
$k=150$	1.00	0.96	0.88	0.86	0.82	0.84	0.86	0.88	0.89	0.92	0.95	0.96	0.97	0.98	1.00

Table B: RV coefficient matrix describing similarities of MPQ distances with different values of r and EKS distances with different values of k for CLR-transformed data.

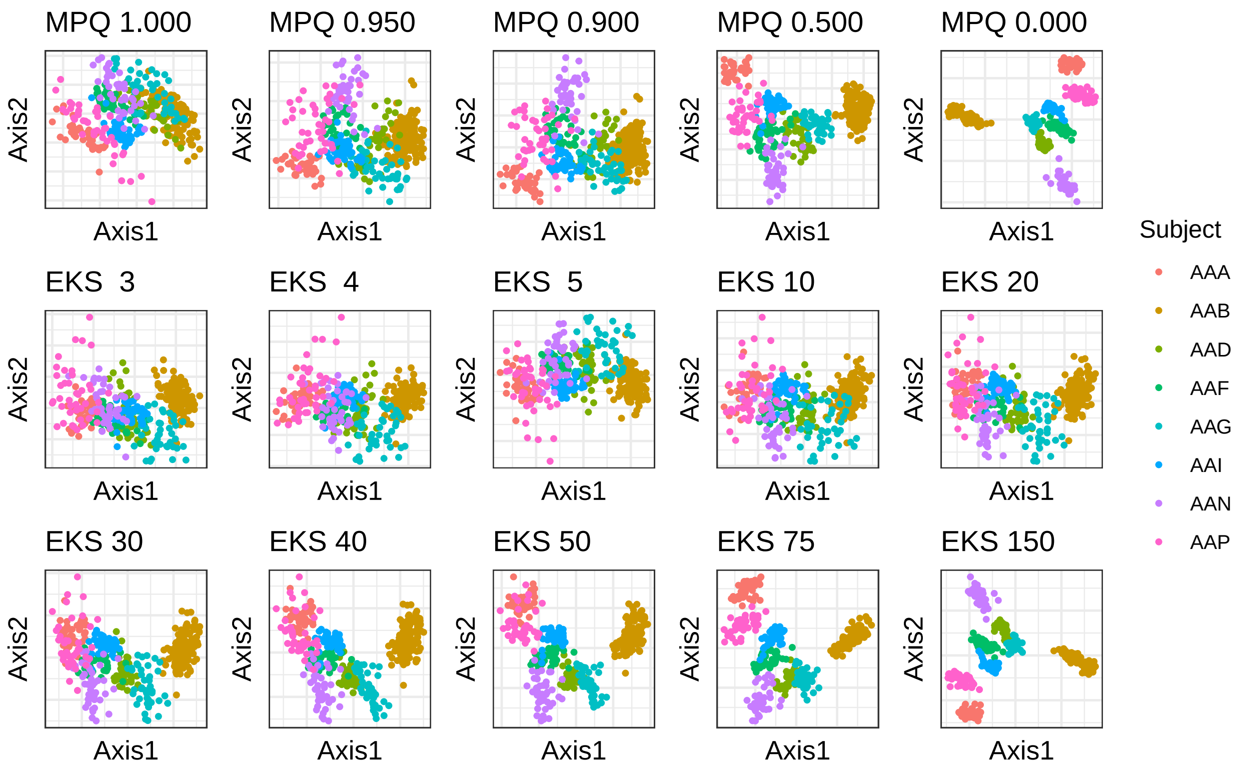

Fig D: Embedding results for MPQ and EKS distances with different values of r and k on the CLR-transformed data.

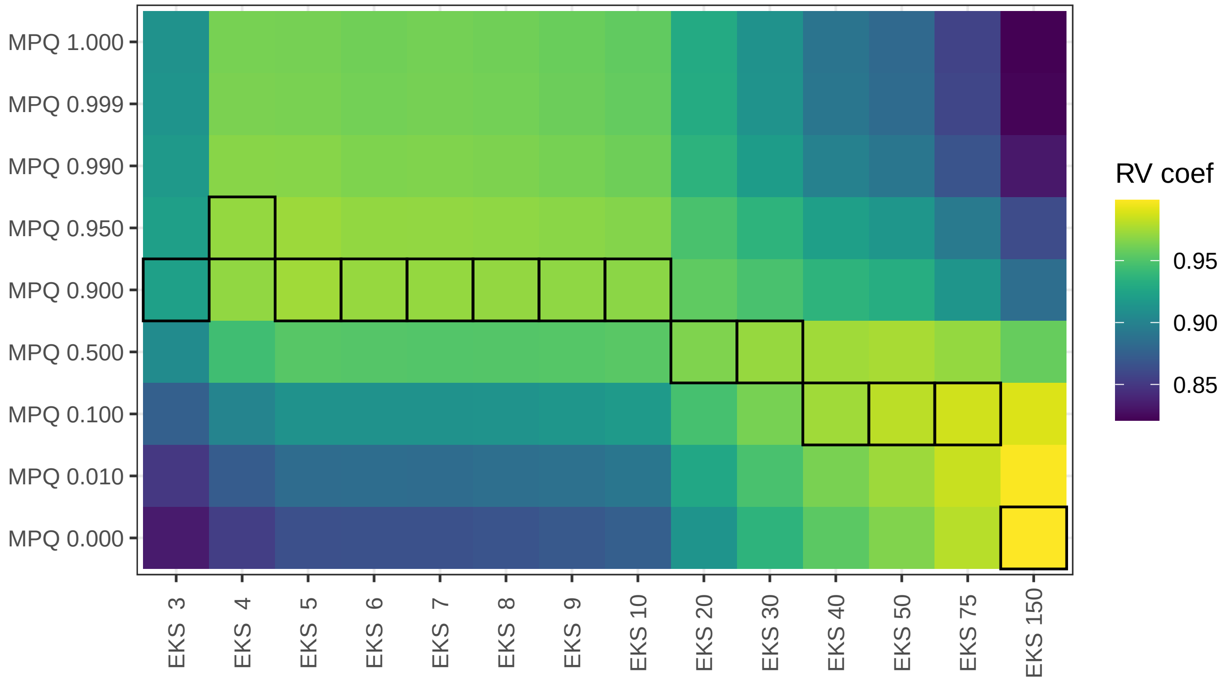

Fig E: Visualization of the RV coefficient matrix describing similarities of different MPQ ad EKS distances for CLR-transformed data. Outlined box shows the MPQ distance that is the most similar to a given EKS distance.

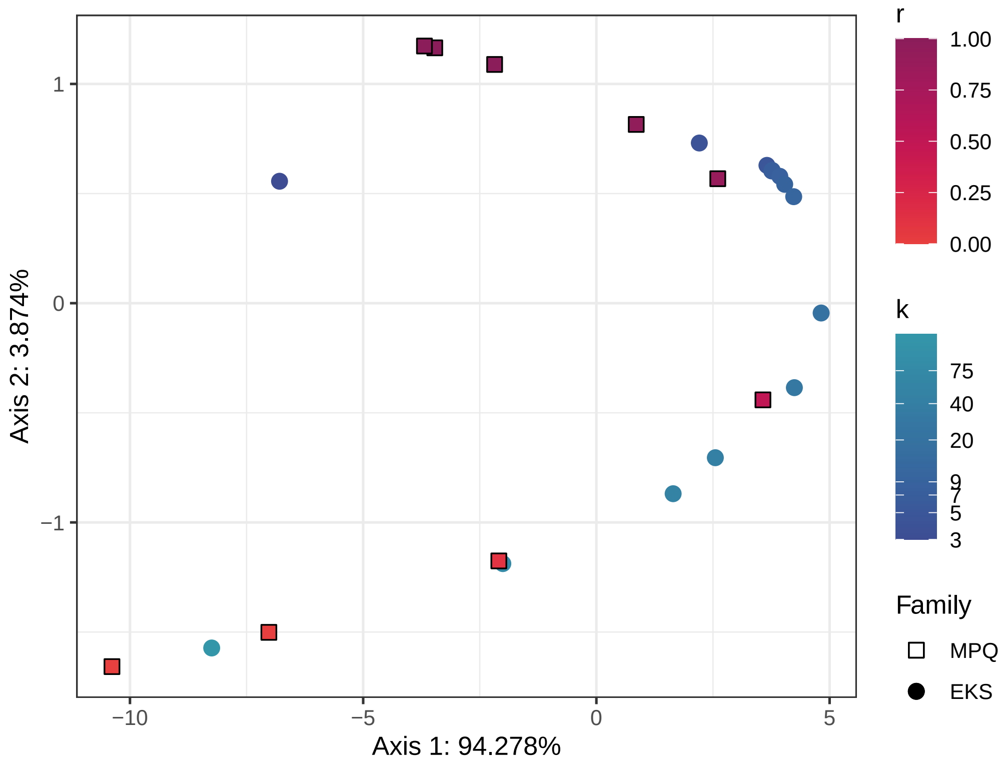

Fig F: DISTATIS representation of the different MPQ and EKS distances using CLR-transformed data. Squares represent EKS distances, circles represent MPQ distances. Darker colors correspond to small values of r/large values of k, and light colors correspond to large values of r/small values of k.

Run Accession	Sample Type	Participant	Sweetener
ERR10082679	Stool sample	A103	Aspartame
ERR10082717	Stool sample	A102	Aspartame
ERR10082766	Stool sample	A102	Aspartame
ERR10082806	Stool sample	A104	Aspartame
ERR10082834	Stool sample	A101	Aspartame
ERR10082872	Stool sample	A102	Aspartame
ERR10082874	Stool sample	A102	Aspartame
ERR10082884	Stool sample	A101	Aspartame
ERR10082910	Stool sample	A104	Aspartame
ERR10082990	Stool sample	A102	Aspartame
ERR10083000	Stool sample	A101	Aspartame
ERR10083006	Stool sample	A103	Aspartame
ERR10083008	Stool sample	A102	Aspartame
ERR10083027	Stool sample	A101	Aspartame
ERR10083036	Stool sample	A101	Aspartame
ERR10083080	Stool sample	A101	Aspartame
ERR10083101	Stool sample	A101	Aspartame
ERR10083108	Stool sample	A101	Aspartame
ERR10083185	Stool sample	A103	Aspartame
ERR10083186	Stool sample	A103	Aspartame
ERR10083202	Stool sample	A101	Aspartame
ERR10083215	Stool sample	A103	Aspartame
ERR10083218	Stool sample	A101	Aspartame
ERR10083228	Stool sample	A103	Aspartame
ERR10083238	Stool sample	A102	Aspartame
ERR10083247	Stool sample	A103	Aspartame
ERR10083252	Stool sample	A102	Aspartame
ERR10083253	Stool sample	A103	Aspartame
ERR10083258	Stool sample	A103	Aspartame
ERR10083366	Stool sample	N600	Control
ERR10083419	Stool sample	N601	Control
ERR10083439	Stool sample	N601	Control
ERR10083455	Stool sample	N602	Control
ERR10083457	Stool sample	N600	Control
ERR10083459	Stool sample	N601	Control
ERR10083471	Stool sample	N600	Control
ERR10083473	Stool sample	N601	Control
ERR10083478	Stool sample	N602	Control
ERR10083484	Stool sample	N600	Control
ERR10083490	Stool sample	N602	Control
ERR10083491	Stool sample	N601	Control
ERR10083493	Stool sample	N601	Control
ERR10083494	Stool sample	N600	Control
ERR10083495	Stool sample	N600	Control
ERR10083498	Stool sample	N601	Control
ERR10083500	Stool sample	N602	Control
ERR10083510	Stool sample	N602	Control
ERR10083511	Stool sample	N602	Control
ERR10083519	Stool sample	N601	Control
ERR10083523	Stool sample	N600	Control
ERR10083524	Stool sample	N600	Control
ERR10083530	Stool sample	N602	Control
ERR10083540	Stool sample	N602	Control
ERR10083541	Stool sample	N600	Control
ERR10083542	Stool sample	N600	Control
ERR10083544	Stool sample	N602	Control
ERR10083552	Stool sample	N601	Control
ERR10083559	Stool sample	N601	Control
ERR10083564	Stool sample	N602	Control
ERR10083774	Stool sample	A103	Aspartame

Table C: Accessions and metadata for the samples used for the non-nutritive sweetener analysis.

References

1. Jukes T, Cantor CR. Evolution of protein molecules. In: Munro H, editor. Mammalian Protein Metabolism. New York, USA: Academic Press; 1969. p. 21–132.
